# Phage (cocktail)-antibiotic synergism: a new frontier in addressing *Klebsiella pneumoniae* resistance

**DOI:** 10.3389/fmicb.2025.1588472

**Published:** 2025-05-07

**Authors:** Chandrasekar Karthika, Nambiraman Malligarjunan, Nagaiah Hari Prasath, Shunmugiah Karutha Pandian, Shanmugaraj Gowrishankar

**Affiliations:** Department of Biotechnology, Science Campus, Alagappa University, Karaikudi, Tamil Nadu, India

**Keywords:** phage therapy, *Klebsiella pneumoniae*, biofilm, cocktail phages antibiotic synergism, jumbo phage, whole genome sequencing, *Galleria mellonella* histopathology

## Abstract

Phages, which play a crucial role in regulating bacterial populations and evolution, have gained renewed attention as potential therapeutic agents especially in the face of rising antimicrobial resistance, such as in *Klebsiella pneumoniae*– a MDR pathogen with significant clinical implications for immunocompromised individuals. In this milieu, the present investigation aimed at evaluating the therapeutic potential of two lytic phages, KPKp (jumbo phage) and KSKp, as potential candidates for phage treatment. Initial purification and TEM characterization revealed their family as *Ackermannviridae* (KPKp) and *Straboviridae* (KSKp). The one-step growth curve analysis divulged that KPKp and KSKp exhibit burst sizes of ~98 and ~121 and latency periods of 8 and 12 min, respectively. Genomic analysis unveiled linear double-stranded DNA as their genome with sizes 206,819 bp (KPKp) and 167,101 bp (KSKp) lacking virulence or lysogenic genes, signifying their therapeutic suitability. Evaluation of phages as a cocktail demonstrated a substantial improvement in lytic ability, achieving complete (100%) lysis (at MOI 1) of clinical isolates compared to individual phages, achieving 50 and 25% lysis at MOI 1. *In vitro* investigations demonstrated that the phage cocktail significantly decreased both planktonic and sessile cells. Additionally, the phage (cocktail)-antibiotic synergism (PAS) achieves over 90% inhibition of *K. pneumoniae*, even at sub-lethal antibiotic doses. PAS treatment significantly prolongs the lifespan of *K. pneumoniae*-infected *Galleria mellonella*. Compared to cocktail phage therapy, PAS demonstrates a superior reduction in bacterial load. In conclusion, the combination of phages and antibiotic holds potential for addressing clinical challenges associated with MDR *K. pneumoniae* infection.

## 1 Introduction

Carbapenem-resistant *Klebsiella pneumoniae* strains, recognized as superbugs, are considered pivotal nosocomial pathogens worldwide, capable of inducing severe infections associated with increased morbidity and mortality rates (Pu et al., 2023). Over the last decade, the global incidence of carbapenem-resistant *K. pneumoniae* infections has witnessed a substantial surge, presenting a serious cause for concern within healthcare systems globally (Tesfa et al., 2022). The resistance of *K. pneumoniae* to carbapenems is linked to the production of remarkably potent carbapenemases and a myriad of other resistance mechanisms, including alterations in cell membrane structure and beta-lactamase activity, all contributing to the formidable challenge of carbapenem resistance (Ho et al., 2019). Notably, carbapenem-resistant *K. pneumoniae* isolates typically exhibit multidrug resistance (MDR), extending beyond carbapenems to include resistance against penicillins, third-generation cephalosporins, fluoroquinolones and aminoglycosides (Ramadan et al., 2022; Zhao et al., 2024). The emergence of MDR in *K. pneumoniae* further exacerbates the urgency of the situation, given the limited availability of alternative treatment options, thus emphasizing the critical need for effective therapeutic interventions against this superbug (Moradigaravand et al., 2017).

The stagnation in the development of new antibiotics over the last two decades has shifted focus toward bacteriophage therapy as a potential solution for MDR bacterial infections (Nikolich and Filippov, 2020). Western countries are reassessing their regulations on phage therapy due to its approach in treating MDR bacterial infections (Venturelli, 2022). Phage therapy offers advantages over antibiotics, such as a narrower antibacterial spectrum, a safer profile and the ability to target bacteria resistant to antibiotics (Romero-Calle et al., 2019). However, a major drawback of phage therapy, similar to antibiotics, is the rapid development of phage resistance by bacteria (Örmälä and Jalasvuori, 2013). The restricted spectrum of antibacterial activity associated with phage treatment poses significant challenges in clinical settings, necessitating swift therapeutic options (Malik et al., 2021). Consequently, combining phages with antibiotics may offer a more effective approach to treating bacterial infections compared to phage therapy.

The synergy between cocktail phages and ciprofloxacin (CIP) is crucial in combating *K. pneumoniae* due to their complementary mechanisms of action (Shariati et al., 2022). CIP works by targeting a bacterial enzyme called gyrase, which is crucial for bacterial DNA replication (Shariati et al., 2022). Its small size allows it to penetrate biofilms, which are protective layers formed by bacteria and effectively stop the growth of bacteria living within the biofilm (Mi et al., 2018). In addition to CIP’s action, phages directly target and kill bacterial cells, enhancing the overall efficacy of treatment against *K. pneumoniae* infections (Gordillo Altamirano et al., 2022). Combining antibiotic with phages reduces host toxicity, improves efficacy and lowers resistance development (Li et al., 2021). Understanding how these combinations affect bacterial metabolism and resistance is essential, especially for addressing carbapenem-resistant *K. pneumoniae*, an area lacking sufficient research attention.

In this study, the synergistic killing of a cocktail of phages in combination with CIP was investigated against carbapenem-resistant MDR *K. pneumoniae* ATCC (American Type Culture Collection) 700603. This strain contains the blaKPC gene, which encodes the *K. pneumoniae* carbapenemase enzyme, responsible for conferring resistance to carbapenem antibiotics. This resistance poses a significant challenge in treating infections caused by the strain (Ghasemnejad et al., 2019). The study utilized two lytic cocktail phages (KPKp and KSKp) at very least multiplicity of infection (MOI) demonstrating over 90% inhibition of *K. pneumoniae*, even at sub-lethal antibiotic doses. These findings suggest that combining phages and antibiotics could be a promising strategy against MDR *K. pneumoniae* infections, offering hope for improved treatment outcomes. By employing the *Galleria mellonella* larvae model to assess *in vivo* effectiveness, this research aims to provide valuable insights into therapeutic applications.

## 2 Materials and methods

### 2.1 Bacterial strains and culture conditions

The reference bacterial strain *K. pneumoniae* 700,603 from the ATCC was used in the study, along with four clinical isolates of *K. pneumoniae*. The GenBank accession numbers are KP CI 1 (MW600514), KP CI 2 (MW600515), KP CI 3 (MW600516), and KP CI 4 (MW600517).

An ORBITEK LEBT-D-1 L orbital shaker was used to grow all bacterial cultures in tryptic soy broth (TSB) overnight at 37°C with agitation at 130 rpm.

For further studies, a 100 μL (1 × 10^8^ CFU/mL) logarithmic phase bacterial suspension (OD600 = 0.2, measured using the SpectraMax M3 instrument) was employed. These included host range identification, planktonic cell lysis testing, temperature and pH stability assessments, mature biofilm destruction, adsorption studies, one-step growth curve analysis, and *in vivo* evaluations.

Pure bacterial cultures grown in TSB were preserved for long-term storage by preparing 25% glycerol stocks and storing them at −80°C.

### 2.2 Assessment of antibiotic susceptibility

All *K. pneumoniae* strains were subjected to antibiotic susceptibility testing using the Kirby Bauer disc diffusion technique (Table 1) in accordance with CLSI standards (CLSI, 2022).

**Table 1:** Zone diameters are measured in antimicrobial disk assays against *K. pneumoniae* ATCC 700603.

S. No.	Classes of antibiotics	Antibiotics	Disk content (mcg)	Sensitive (mm or more)	Intermediate (mm)	Resistant (mm or less)	Sensitivity
1	Carbapenems	Meropenem	10	23	20–22	19	R
2	Carbapenems	Imipenem	10	23	20–22	19	R
3	Cephalosporins	Cefepime	30	25	19–24	18	R
4	Cephalosporins	Ceftriaxone	30	23	20–22	19	R
5	Cephalosporins	Ceftazidime	10	22	19–21	19	R
6	Monobactams	Aztreonam	30	21	18–20	17	R
7	Fluoroquinolones	Ciprofloxacin	5	26	22–25	21	R
8	Fluoroquinolones	Levofloxacin	5	21	17–20	16	R
9	Aminoglycosides	Gentamicin	10	15	13–14	12	R

Mcg, Microgram; mm, millimeter; CLSI, Clinical and Laboratory Standards Institute; ATCC, American Type Culture Collection; R, Resistant.

In brief, cultures in the logarithmic phase were spread onto Mueller Hinton Agar (MHA) plates, which were then placed with antibiotic discs. Following an 18 h incubation period at 37° C, the resulting inhibitory zones were compared to a standard chart.

Commercially available antibiotic discs, including Meropenem (10 μg/mL), Imipenem (10 μg/mL), Cefepime (30 μg/mL), Ceftriaxone (30 μg/mL), Ceftazidime (10 μg/mL), Aztreonam (30 μg/mL), CIP (5 μg/mL), Levofloxacin (5 μg/mL) and Gentamicin (10 μg/mL) were obtained from HiMedia in India.

### 2.3 Characterization of phenotypic traits

#### 2.3.1 Isolation, purification, and quantification of phages

Phages infecting *K. pneumoniae* were isolated following the protocol described by Karthika et al. (2023). Two lytic phages, KPKp and KSKp, were isolated, respectively, from pond and sewage samples collected near Karaikudi, Sivaganga District, Tamil Nadu. In order to isolate the bacteria, 0.5 mL of *K. pneumoniae* ATCC 700603, 4 mL of the environment sample, and 0.5 mL of 10X TSB broth were cultured for 24 h at 37°C. An ESCO centrifuge was used to centrifugal the mixture for 10 min at 37°C at 7910 × *g*. To get rid of the microbes, the supernatant was filtered across a hydrophilic polyethersulfone (PES) membrane with a pore size of 0.22 μm. Phage presence was confirmed by a spot test on tryptic soya agar (TSA) inoculated with logarithmic-phase bacterial culture, with lysis zones indicating phage lytic activity. After being gathered in sterile sodium chloride-magnesium sulphate (SM) buffer (pH 7.5), the phages were treated with 0.5% chloroform, centrifuged, and filtered to obtain the phage filtrate.

Phage purification was performed using the Double Layer Agar (DLA) technique (Lewis et al., 2020), with iterative plaque purification until uniform plaques were obtained. Purified phages were multiplied by infecting *K. pneumoniae* cells in the early exponential growth phase, followed by centrifugation and filtration, and stored at 4°C.

The DLA test, which was based on Zhou et al. (2022), was used to quantify phages. After incubating at 37°C for 16 h, plaques from repeated dilutions of the phage stock were counted on plates.

Phage titers were obtained using the following formula in plaque-forming units PFU/mL = Plaque Count × Dilution Factor/Volume (mL).

PFU to colony-forming units (CFU) of the host bacterium was used to calculate the MOI.

#### 2.3.2 Evaluation of the host’s range and efficiency of plating (EOP)

The acquired phages were evaluated for pathogenicity using the spot assay method against a variety of common Gram-positive and Gram-negative bacterial pathogens (Table 2), as well as four clinical MDR *K. pneumoniae* isolates (Table 3). The procedure was adapted from a prior study by Li et al. (2016) with minor modifications. TSA plates were inoculated with one hundred microliters of rapidly multiplying clinical and reference strain *K. pneumoniae* cultures (~10^8^ CFU/mL), followed by spotting 10 μL (~10^8^ PFU/mL) of individual or cocktail phage stocks.

**Table 2:** Different bacterial strains employed in spot test assay for cocktail (KPKp and KSKp) phages (Supplementary Figure 1A).

S. No	Bacterial strain	Phage activity (+/−) (cocktail phages)
1	*Klebsiella pneumoniae* ATCC 700603	+
2	*Klebsiella aerogenes* ATCC 35029	−
3	*Pseudomonas aeruginosa* ATCC PA01	−
4	*Methicillin resistant Staphylococcus aureus* ATCC 33591	−
5	*Acinetobacter baumannii* ATCC 19606	−
6	*Enterococcus faecium* ATCC 51299	−
7	*Acinetobacter baumannii* ATCC 19606	−
8	*Enterococcus faecium* ATCC 51299	−
9	*Escherichia coli* MTCC	−
10	*Salmonella Typhi* MTCC 733	−

**Table 3:** Results of the EOP test and spot test assay for different *K. pneumoniae* clinical isolates compared with *K. pneumoniae* ATCC 700603 (Supplementary Figures 1B–D).

S. No	Bacterial strains	Susceptible to phage (Spot test)			Efficiency of plating (EOP)		
		KPKp	KSKp	Cocktail	KPKp	KSKp	Cocktail
1	KPCI1	+	−	+	0.64	0.026	0.85
2	KPCI2	−	+	+	0.05	6.78	0.90
3	KPCI3	−	0	0	0	0.26	0.33
4	KPCI4	+	0	+	0.55	0.0002	0.70

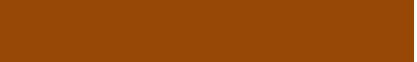
 Highly virulent (≥ 0.5); 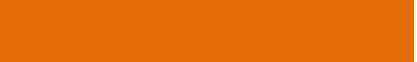 Moderately virulent (≥ 0.1 to < 0.5); 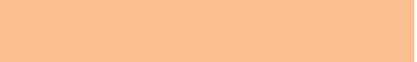 Low virulent (≥ 0.001 to < 0.1); 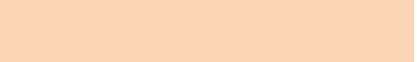 Inefficiently virulent (< 0.001); 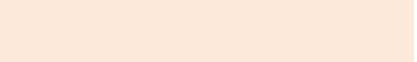 Non-virulent 0.

After 16 h of 37°C incubation, lysis zones formed at areas where the phages were identified on the plates. Each phage’s efficacy against many bacterial strains was evaluated using triplicate trials. The resulting lysis patterns were categorized as susceptible (+), moderately susceptible (0), or resistant (−). The DLA technique was used to evaluate phage efficacy against a range of clinical isolates.

The phages exhibiting the broadest host range, as determined by the spot assay, were further evaluated using the EOP assay. Phage (KPKp, KSKp, and their cocktail) lysis activity against clinical isolates was assessed using DLA technique. A series of tenfold dilutions of each phage (KPKp, KSKp, and the cocktail) were prepared in SM buffer, ranging from 10^6^ to 10^9^, and each dilution was tested in triplicate. For the EOP assay, 100 μL of log-phase *K. pneumoniae* clinical isolate culture was mixed with 100 μL of the corresponding phage dilution and incubated for 16 h at 37°C. Following incubation, the mixtures were immediately subjected to the DLA assay, and PFUs were counted. If no plaques were observed at the 10^6^ dilution, lower dilutions were tested.

Clinical *K. pneumoniae* isolates’ PFU was divided by the reference strain’s PFU (*K. pneumoniae* ATCC 700603) to determine EOP. The phages were classified as inefficiently virulent (EOP < 0.001) or non-virulent (no plaques were seen), low virulence (EOP ≥ 0.001 and < 0.1), moderately virulent (EOP ≥ 0.1 and < 0.5), and highly virulent (EOP ≥ 0.5) based on the average EOP values.

#### 2.3.3 Transmission electron microscopy (TEM)

With the necessary modifications, the TEM approach described by Zhou et al. (2022) was used to investigate the morphological traits of pure phages. Following purification, 2% uranyl acetate was utilized for negative staining, and the phages were dropped upon carbon-covered 300-mesh copper grids (Sigma-Aldrich, USA). Using a TEM running at 60 kV, micrographs were produced.

#### 2.3.4 Assessment of adsorption and one-step growth curve rate

The adsorption rate of KPKp & KSKp phages and the kinetics of one-step growth were determined according to Sharma et al. (2021) using a modified version of the approaches. The host bacterial culture was exposed to phage suspensions of KPKp & KSKp, each with a MOI of 0.001, at a concentration of ~10^8^ CFU/mL in order to estimate the adsorption rate. At 37°C, the mixture was thereafter incubated. The titers of non-adsorbed phages were determined by centrifuging samples that were aseptically collected every two min for 20 min, followed by the DLA method.

The one-step growth curve experiment involved growing the host bacterial cells in 10 mL of TSB medium to a density of ~10^8^ CFU/mL. At a MOI of 0.001, a phage suspension was introduced. Samples were taken every 10 min for 80 min following a 5 min incubation, and supernatants containing unbound phages were removed. Using the DLA approach, the quantity of released phages at each time point was measured. Calculated the delay and burst size using the below formula.


$$\begin{array}{l} \mathrm{Burst size}=\mathrm{the initial number of infectious bacterial cells}/\\ \mathrm{Phage titers during the plateau period} \end{array}$$

#### 2.3.5 Assessment of stability across different pH and temperature conditions

With some slight modifications, the effects of temperature and pH on the pathogenic efficacy of isolated phages were examined using the methods described by Sharma et al. (2021). Incubation was conducted for 24 h at temperatures ranging from 4 to 70°C using phage lysates (1.1×10^8^ PFU/mL). The lysates were then processed by DLA, and phage titers were ascertained using this technique. Three separate assessments were made of both phages’ temperature stability.

Before introducing phage suspensions (0.1 mL of KPKp & KSKp each), the pH of the SM buffer was adjusted with one N HCl and NaOH. The combination was then incubated for 24 h at 37°C to assess pH stability. The DLA method was used to assess phage activity and stability studies. This test was performed independently and in triplicate on the KPKp and KSKp phages. The results, presented as mean ± standard deviation, were analyzed for statistical significance using appropriate tests such as ANOVA to compare phage titers across different conditions.

### 2.4 Revealing the genomic features

#### 2.4.1 Phage DNA isolation and sequencing

Concentrated phage lysates were digested with Proteinase K (55°C, overnight) to break down capsid proteins after being treated with DNase I (37°C, 30 min) to eliminate host DNA. After centrifuging at 12,000 × *g* for 15 min, the aqueous phase was recovered from the DNA extraction process, which used phenol:chloroform:isoamyl alcohol (25:24:1) (Sambrook and Russell, 2001). After being precipitated using cold ethanol and sodium acetate, the genomic DNA was rinsed with 70% ethanol, allowed to air dry, and then reconstituted in water devoid of nuclease. Whole genome sequencing (WGS) was used to the isolated DNA. An Illumina HiSeq 2,500 sequencing apparatus was used for WGS. Platanus-allee carried out *de novo* assembly. The Artemis program was used to predict phage genome open reading frames (ORFs) with a minimum protein length of 30 amino acids. These predicted ORFs were then compared to non-redundant protein sequence databases using BLAST at NCBI, with E-values set at less than 0.2. RNAmmer (v1.2) and tRNAscan-SE (v1.23) were used to identify ribosomal RNAs and transfer RNAs, respectively. While Megablast at NCBI was used to assess the sequence homology of entire viral nucleotides among closely related phages, the BDGP prediction engine was used to identify bacterial promoters. Pyani 0.2.10, the average_nucleotide_identity.py script, and the ANIm technique were used to conduct the phylogenetic studies. Genomic comparisons between the sequenced phage genome and similar phage genomes were carried out using Easyfig Software version 2.1.

### 2.5 *In vitro* therapy assessment

#### 2.5.1 Minimal inhibitory concentration (MIC)

In 24-well microtiter plates (MTPs) using the broth microdilution method, the MIC of antibiotics against *K. pneumoniae* ATCC 700603 was determined in accordance with the Gordillo Altamirano et al. (2022) methodology. Initially, a 1% inoculum (1 × 10^8^ CFU/mL) was introduced into the wells of the MTPs. The 24-well MTPs were filled with an equivalent volume of broth treated with antibiotics at various concentrations (from 1 to 1,024 μg/mL), with 1 mL of broth supplied to each well. The antibiotics utilized in the study are detailed in Table 4. MTPs were incubated at 37°C for 16 h with untreated bacteria as controls. Experiments were performed in triplicate and independently repeated thrice.

**Table 4:** The MIC of antibiotics against *K. pneumoniae* ATCC 700603 was determined using the micro broth dilution assay.

S. No.	Antibiotics	Sensitive (≤S)	Intermediate (I)	Resistant (≥R)	MIC (*μ*g/mL)
1	Meropenem	1	2	4	16
2	Imipenem	1	2	4	16
3	Cefepime	2	4–8	16	64
4	Ceftriaxone	1	2	4	64
5	Ceftazidime	4	8	16	64
6	Aztreonam	4	8	16	32
7	Ciprofloxacin	0.25	0.5	1	4
8	Levofloxacin	0.5	1	2	8
9	Gentamicin	4	8	16	16

MIC, minimum inhibitory concentrations; μg, microgram; mL, milliliter.

#### 2.5.2 *In vitro* phage-antibiotic synergy (PAS) experiments

With a few small modifications, the approach described by Gordillo Altamirano et al. (2022) was used to investigate the synergistic antibacterial impact of phages and antibiotics. A 48-well MTPs was used, with each well containing an initial inoculum of 1 × 10^8^ CFU/mL. Each well followed a checkerboard pattern of phage concentrations (KPKp ranging from MOI 10 to 0.001, KSKp ranging from MOI 10 to 0.001, and cocktail phages ranging from MOI 0.1 to 0.00001, rows) along with CIP concentrations (ranging from 4 to 0.25 μg/mL, columns). Control wells included vehicle controls and bacteria-only controls in the initial concentration row and column. MIC determination was performed after 24 h incubation at 37°C by measuring OD_600_ using a SpectraMax 3 spectrophotometer. Synergistic interactions were evaluated from triplicate OD readings for each phage-antibiotic combination.

The term “sub-lethal antibiotic doses” refers to CIP concentrations lower than the MIC value determined for *K. pneumoniae* ATCC 700603 (MIC = 4 μg/mL). For PAS experiments, concentrations of 1 μg/mL and 0.5 μg/mL were used, corresponding to 4-fold and 8-fold reductions from the MIC, respectively. These concentrations were selected based on their inability to inhibit bacterial growth when used alone but demonstrated synergistic activity in combination with phages.

#### 2.5.3 Assessment of planktonic cell lysis

Phage-mediated lysis of *K. pneumoniae* planktonic cells was assessed using a modified method based on Kim et al. (2019). A 1% overnight culture of *K. pneumoniae* was diluted in TSB and grown to mid-log phase (OD_600_ = 0.2) at 37°C. Cultures were treated with phage KPKp, KSKp, or their combination at MOIs of 10, 1, and 0.1. Additional treatments included CIP (0.5 μg/mL), cocktail phages (MOI 0.001), and PAS (cocktail phages at MOI 0.001 + CIP at 0.5 μg/mL).

Turbidity was measured hourly for 24 h at 600 nm. CFU enumeration was performed at selected intervals by serial dilution in 0.85% sterile PBS, followed by plating on TSA and incubation.

Results are presented as mean ± SD from triplicate experiments. Time-kill kinetics, including OD and CFU data, were analyzed for statistical significance using one-way ANOVA.

#### 2.5.4 Phage induced disruption of mature biofilms

A modified protocol based on Kim et al. (2019) was used to evaluate the ability of phages to disrupt *K. pneumoniae* biofilms. Two sets of test tubes containing TSB and 1% mid-log phase *K. pneumoniae* were prepared one for CV staining and the other for CFU/PFU enumeration. After 24 h incubation at 37°C, non-adherent planktonic cells were removed by rinsing with sterile 0.85% PBS.

Adherent biofilms were treated with CIP (4 μg/mL), individual phages (MOI 10), a phage cocktail (MOI 10), or PAS (cocktail phages at MOI 0.001 + CIP at 0.5 μg/mL), followed by the addition of fresh TSB. Untreated biofilms served as controls.

For PFU/CFU quantification, supernatants from the first set of tubes were centrifuged (9,180 × *g*, 5 min), and PFU was determined using the DLA method. After two PBS washes, adherent biofilm cells were resuspended by vortexing for CFU analysis.

Biofilm biomass in the second set was quantified via 0.4% CV staining. After 15 min, excess dye was rinsed off, and stained tubes were air-dried. Bound dye was solubilized in 15% glacial acetic acid, and absorbance was recorded at 570 nm. Biofilm biomass (%) was calculated as: [(OD₅₇₀ treated) / (OD₅₇₀ control)] × 100.

All quantitative data, including biomass, CFU/PFU, and metabolic activity (fluorescence intensity), are expressed as mean ± SD from three independent experiments. Statistical differences were assessed using ANOVA or unpaired t-tests.

##### 2.5.4.1 Resazurin based metabolic activity assay

The impact of phage therapy on *K. pneumoniae* biofilm viability over time was assessed using a resazurin-based assay as described by Costa et al. (2021). After 24 h of biofilm development, planktonic cells were removed from biofilms, and wells were gently rinsed with sterile 0.85% PBS.

Biofilms were then treated with CIP at its MIC (4 μg/mL), individual phages (KPKp and KSKp, MOI 10), a phage cocktail (MOI 10), or a PAS combination (cocktail phages at MOI 0.001 and CIP at 0.5 μg/mL). Treated plates were incubated at 37°C for 24 h.

Post-treatment viability was evaluated using Alamar Blue staining. Wells were supplemented with 2 μL of 10 mg/mL Alamar Blue solution and incubated at 37°C for 4 h in the dark. Fluorescence intensity was measured at excitation/emission wavelengths of 530/590 nm using a fluorescence spectrophotometer.

##### 2.5.4.2 Microscopic analysis of *Klebsiella pneumoniae* biofilms

Microscopy was employed to qualitatively assess biofilm architecture and evaluate the impact of phage treatment on the viability and spatial distribution of sessile cells. Three distinct microscopic techniques were used in this study.

Glass slides (1 cm^2^) were placed in each well of a 24-well microtiter plate (Tarsons, India) to support biofilm formation. Wells were inoculated with 1 mL of TSB medium containing 1% bacterial suspension and incubated at 37°C for 24 h. After incubation, planktonic cells were gently removed by rinsing three times with sterile 0.85% PBS, leaving the biofilm intact. Control groups with and without phages or antibiotics were included, and 1 mL of fresh TSB was added to preserve biofilm structure. After the initial 24 h incubation, biofilms were treated with a PAS combination consisting of cocktail phages (MOI 0.001) and CIP (0.5 μg/mL).

###### 2.5.4.2.1 Examination under a light microscope

Glass slides were rinsed with sterile 0.85% PBS, air-dried, and stained with 0.4% crystal violet for 15 min, following the method of Karthika et al. (2024) with minor modifications. Excess stain was removed with sterile PBS, and slides were air-dried before observation under a light microscope at 400 × magnification.

###### 2.5.4.2.2 Fluorescence microscopy examination

Live-dead staining was performed as described by Jaiswal and Mishra (2018). Slides were washed with sterile 0.85% PBS and stained in the dark for 20 min using a 1:1 mixture of 0.1% ethidium bromide (EtBr) and acridine orange (AO). After rinsing with sterile PBS, slides were air-dried and examined under a fluorescence microscope at 400 × magnification.

###### 2.5.4.2.3 Confocal laser scanning microscope (CLSM)

After staining with 0.1% AO, slides were incubated in the dark for 5 min, rinsed with distilled water to remove excess stain, air-dried, and imaged using a CLSM (LSM 710, Carl Zeiss, Germany).

### 2.6 Evaluation of *in vivo* therapeutic efficacy

#### 2.6.1 A model of infectious disease based on *Galleria mellonella* larvae

The therapeutic effectiveness of lytic phages identified against *K. pneumoniae*, specifically KPKp and KSKp, both alone and in combination as a cocktail, as well as in treating PAS, was assessed using *G. mellonella* larvae in an *in vivo* model of *K. pneumoniae* infection.

Following previously published guidelines by Jeon et al. (2019) with slight modifications, the larvae were reared on a synthetic food consisting of 20% glycerol, 20% maize flour, 15% milk powder, 15% wheat flour, 5% dry yeast, and 25% liquid honey, while maintaining a constant temperature of 37°C.

Larvae were acclimated in 90-mm Petri dishes at 37°C for 24 h without food and in complete darkness. Prior to injection, 200–250 mg larvae were surface-sterilized with 70% ethanol to minimize contamination risk. After this, the larvae were split up into 17 experimental groups, including Control groups: (1) Naive control, (2) PBS alone, (3) SM buffer alone, (4) distilled water with few drops of HCl (CIP diluent), (5) KPKp alone, (6) KSKp alone, Infection control group: (7) *K. pneumoniae* infection alone, Treatment groups: (8–10) KPKp phage treatments at MOIs of 0.1, 1, and 10, 11–13, KSKp phage treatments at MOIs of 0.1, 1, and 10, 14–16, Cocktail treatments (KPKp & KSKp phages) at MOIs of 0.1, 1, and 10, 17, PAS treatment combining cocktail phages (MOI 0.001) with CIP (0.5 μg/mL).

To initiate infection, 10 μL of saline or bacterial suspension was injected into the right proleg, and 10 μL of antibiotic, buffer, or phages into the left. Infected larvae were incubated in 90-mm Petri dishes at 37°C in the dark. Larvae showing movement without stimulation and no melanization were recorded as alive. Survival was monitored every 24 h over 120 h. In contrast, larvae that displayed neither melanization nor movement were classified as dead. Upon experiment termination, larvae from each group underwent homogenization and were subjected to microbial burden assessment through culturing on TSA plates for CFU enumeration. Each experiment was repeated three times, and survival percentages at different time points were recorded and analyzed. Larval survival data from these triplicate experiments were plotted using the Kaplan–Meier method, and statistical significance between survival curves was assessed using the log-rank test. Bacterial burden data (CFU counts), also obtained from triplicate experiments, were analyzed for statistical significance using unpaired t-tests.

#### 2.6.2 Histology of larval tissue: investigating microstructural characteristics

As part of this research, larval specimens were subjected to histological processing, following an adapted protocol based on previous work by Jeon et al. (2019). After being fixed for four days in a 10% neutral buffered formalin solution, the larvae from each experimental group were embedded in paraffin for further examination.

The larvae were cut into longitudinal pieces and allowed to air dry before being examined under a light microscope. After paraffin embedding, tissue sections were stained with hematoxylin for microscopic analysis. This histology approach makes it easier to examine and analyze the larval samples in detail.

### 2.7 Analytical statistics

Biological triplicates were used in each experiment, and at least three technical duplicates were included in each set. Unpaired t-tests and analysis of variance (ANOVA) were both used in the statistical analysis. The mean values were displayed along with the relevant standard deviations. The Kaplan–Meier method was used to examine the survival data and create survival curves. Changes in the survival rates were then evaluated using the log-rank test. Data interpretation and graphical display were done using GraphPad Prism 7.04 (GraphPad Software, Inc., La Jolla, USA). A statistical significance criterion of **** (*p* < 0.0001), *** (*p* < 0.001), ** (*p* < 0.01), and * (*p* < 0.05) was used.

## 3 Results

### 3.1 Phenotypic analysis

#### 3.1.1 Phage isolation and purification

**Figure 1:**
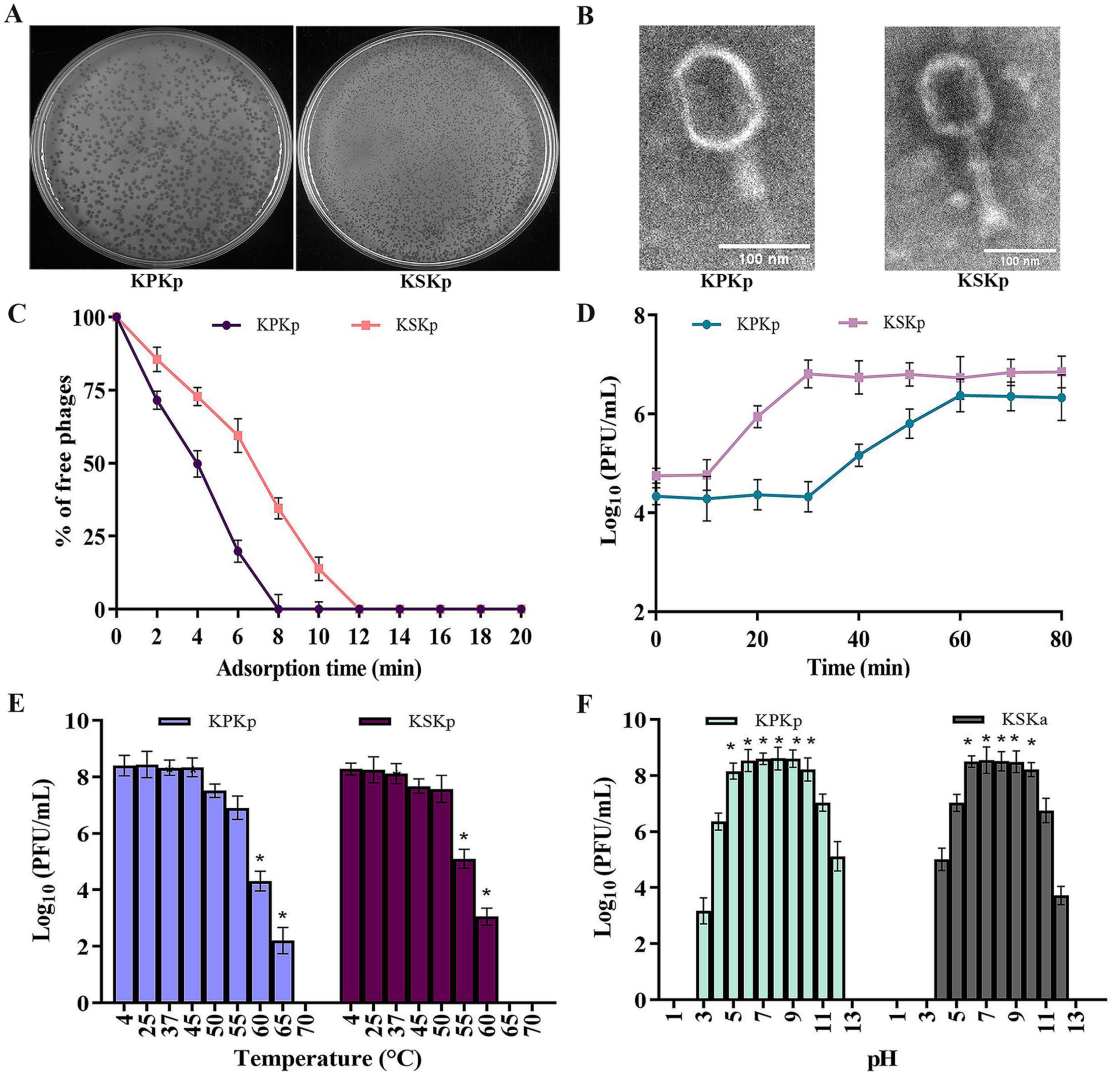
Phenotypic analysis for the KPKp and KSKp phages’ characterization. **(A)** After infected with *K. pneumoniae* ATCC 700603, the KPKp (Left) and KSKp (Right) phages generate homogeneous plaques, according to the results of the soft agar overlay experiment. **(B)** Using a scale bar of 100 nm, TEM displays the morphological traits of the phage families *Straboviridae* (KSKp, Right) and *Ackermannviridae* (KPKp, Left). **(C)** Adsorption rates of KPKp and KSKp phages to *K. pneumoniae* cells are illustrated. **(D)** One-step growth assays illustrate the replication dynamics of KPKp and KSKp phages. **(E)** Thermal stability of KPKp and KSKp phages assessed across various temperatures. **(F)** pH stability profiles of KPKp and KSKp phages assessed at 37°C across different pH conditions. Data represent the mean ± standard deviation from three independent experiments. Asterisks (*) indicate statistically significant differences (*p* < 0.05).

Two highly lytic phages, KPKp and KSKp, were isolated with strong activity against MDR *K. pneumoniae* (ATCC 700603). Spot test analysis confirmed their lytic activity through the formation of clear zones on bacterial lawns. KPKp exhibited moderate plaque formation with a surrounding halo zone, suggesting potential depolymerase activity (Figure 1A). In contrast, KSKp produced plaques without a halo. After several rounds of plaque purification, both phages were concentrated, yielding a final stock concentration of 1.1 × 10^8^ PFU/mL for further experiments (Figure 1A).

Phage cocktails used in the study were prepared by mixing equal multiplicities of infection (MOI 0.1, 1, and 10) of KPKp and KSKp, resulting in a 1:1 ratio optimized for evaluating treatment efficacy against *K. pneumoniae* (ATCC 700603).

#### 3.1.2 Host range and EOP against *Klebsiella pneumoniae*

The cocktail phages’ host range was assessed using ten bacterial strains (Table 2; Supplementary Figure 1A). These included eight Gram-negative and two Gram-positive bacteria. The cocktail phages showed lytic activity specifically against *Klebsiella pneumoniae*, while no activity was observed against the other Gram-negative or Gram-positive strains tested. This indicates the narrow host range and high specificity of the phages toward clinical *K. pneumoniae* isolates (Table 3; Supplementary Figures 1B–D).

KPKp exhibited EOP formation against clinical isolates, with 50% showing high efficiency, 25% moderate efficiency, and 25% low efficiency (Table 3; Supplementary Figure 1B). Similarly, KSKp demonstrated plaque formation efficiency, with 25% high, 25% moderate, 25% low, and 25% inefficient efficiency (Table 3; Supplementary Figure 1C). In comparison, the phage cocktail showed greater efficiency than the individual phages, with 75% high and 25% moderate efficiency (Table 3; Supplementary Figure 1D).

#### 3.1.3 Physical and nomenclatural traits of two phages

TEM, a standard technique for classifying viruses, was used to evaluate the morphological features of the isolated phages. The phages exhibited clear morphological differences in head and tail structures. KPKp displayed an icosahedral head with dimensions ~104.8 ± 17 nm in length and 83.6 ± 10 nm in width, along with a tail measuring 114 ± 1 nm in length and 24.3 ± 5 nm in width, featuring indistinct tail appendages. In contrast, KSKp exhibited an icosahedral head measuring around 110.4 ± 2 nm in length and 89.2 ± 3 nm in width, coupled with a tail spanning 134.6 ± 6 nm in length and 29.5 ± 8 nm in width, adorned with tail spikes at its base (Figure 1B). These structural differences suggest potential variations in phage-host interactions and infection mechanisms.

#### 3.1.4 Phage growth and adsorption kinetics

The adsorption period and one-step growth curve were employed to analyze the infection dynamics of phages. KPKp and KSKp in bacterial cultures. Adsorption kinetics analysis (Figure 1C) revealed similar rates for both phages, with over 99% attachment to host cells occurring in 8 min for KPKp and 12 min for KSKp.

Subsequent examination of the latent period (Figure 1D) demonstrated a 30 min latent period for KPKp and a 10 min latent period for KSKp, with a plateau observed around 60 (KPKp) and 30 (KSKp) min. The phage particle burst sizes for KPKp and KSKp were determined to be 98 and 125 per infected cell, respectively. Understanding the dynamics of infection and the phages’ ability to replicate within the host bacterial culture is aided by these discoveries.

#### 3.1.5 Effects of temperature and pH variations on phage survival and stability

To make sure there was no loss of lytic activity, the infectivity of KPKp and KSKp phages pre-incubated at different pH and temperature levels was evaluated. Phage infectivity decreased significantly, reaching complete loss at 70°C (KPKp) and 65°C (KSKp) (Figure 1E) (*p* < 0.05).

Furthermore, these phages’ stability was investigated during a 24 h period. The infectivity of KPKp and KSKp against *K. pneumoniae* was essentially unaffected by pH values between 6 and 10 (Figure 1F). With some residual stability seen between pH values 3–4 and 11–2, KPKp demonstrated exceptional stability between pH values 5 and 10. Similarly, KSKp retained high stability between pH 6–10, with moderate stability observed between pH 4–5 and 11–12 (*p <* 0.05).

Both phages exhibited stability under moderate pH conditions; however, KPKp demonstrated broader tolerance, particularly at pH 5, indicating better adaptability to gastrointestinal-like environments. KSKp showed greater sensitivity to heat, with loss of infectivity at 65°C, while KPKp remained stable up to 70°C, reflecting superior thermal resilience.

### 3.2 Investigating the genomic characteristics of isolated phages

**Figure 2:**
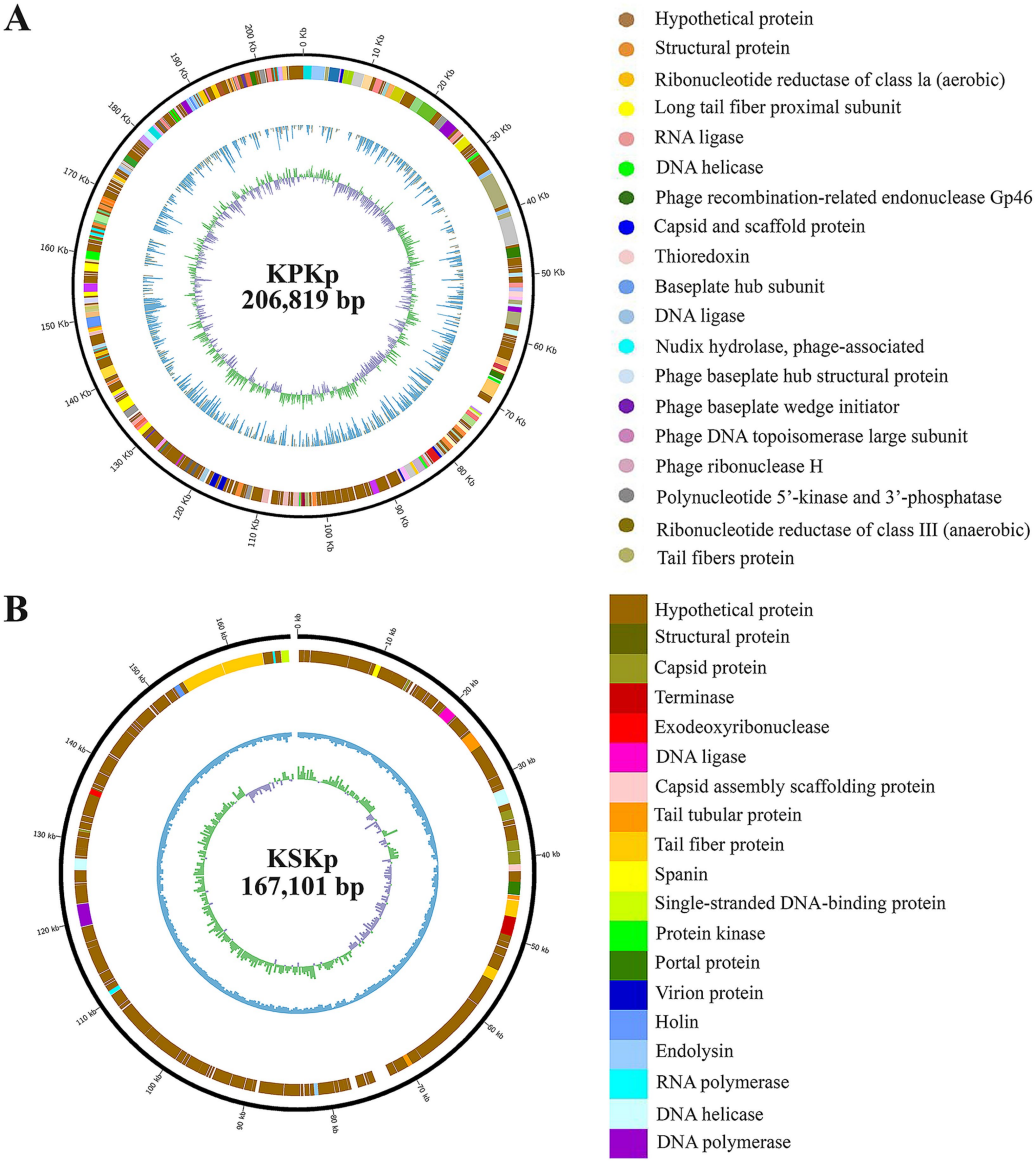
Genomic characterization of KPKp and KSKp Phage. Genome organization maps for KPKp **(A)** and KSKp **(B)**, illustrating the distribution of coding sequences and similar annotations across both phages. Functional annotations, performed using NCBI BLAST and Artemis, reveal essential genes involved in phage biology.

The genomic characteristics of the isolated phages were thoroughly investigated, with Figure 2 and Supplementary Figures 2–4 providing critical insights into their functional potential and evolutionary relationships. Figure 2 presented the genome map of the isolated phages, highlighting essential features such as ORFs and annotated genes.

The WGS analysis of phages KPKp and KSKp revealed distinct genomic characteristics. KPKp (GenBank accession no.: PP791833.1) exhibited a larger genome size of 206,816 bp with a GC content of 39.74%, classifying it as a jumbo phage based on its large genome size. It contained over 485 coding sequences, including 273 sequences with hypothetical functions and 212 sequences with known functions. Additionally, KPKp harbored over 16 tRNA sequences (Figure 2A; Supplementary Table 1).

On the other hand, KSKp (GenBank accession no.: PQ306550.1) had a genome size of 167,101 bp with a GC content of 39.6%. BLAST analysis showed a 98% similarity between KSKp and *Klebsiella* phage vB_KpnM_FRZ284 (MZ602148.1). KSKp contained a total of 296 genes, including essential genes involved in the phage life cycle, such as endolysin and holin. It also harbored 16 tRNA coding genes (Figure 2B; Supplementary Table 1).

Based on genomic features and comparative analyses, KPKp was taxonomically classified under the family *Ackermannviridae* and order *Caudoviricetes* (Zhu et al., 2022), while KSKp was assigned to the family *Straboviridae* within the same order.

Figures 2A,B summarizes the functional predictions of the annotated genes, outlining potential roles in the phage lifecycle. Understanding these roles is crucial for assessing the efficacy of the phage as a therapeutic agent against bacterial infections.

The alignment (Supplementary Figures 2A,B [KPKp, KSKp]) and percentage identity (Supplementary Figures 2C,D [KPKp, KSKp]) results with closely related phages reveal regions of conservation and divergence within the phage genome.

Supplementary Figures 3, 4 present a phylogenetic analysis comparing the isolated phage with previously characterized phages, providing insights into its evolutionary relationships and genomic relatedness within the broader phage taxonomy.

The genomic characterization and functional analysis of the isolated phages offer insights into their genetic composition and potential applications.

### 3.3 *In vitro* characterization of therapeutic phages

#### 3.3.1 Exploring the synergistic interplay of phages and antibiotics

**Figure 3:**
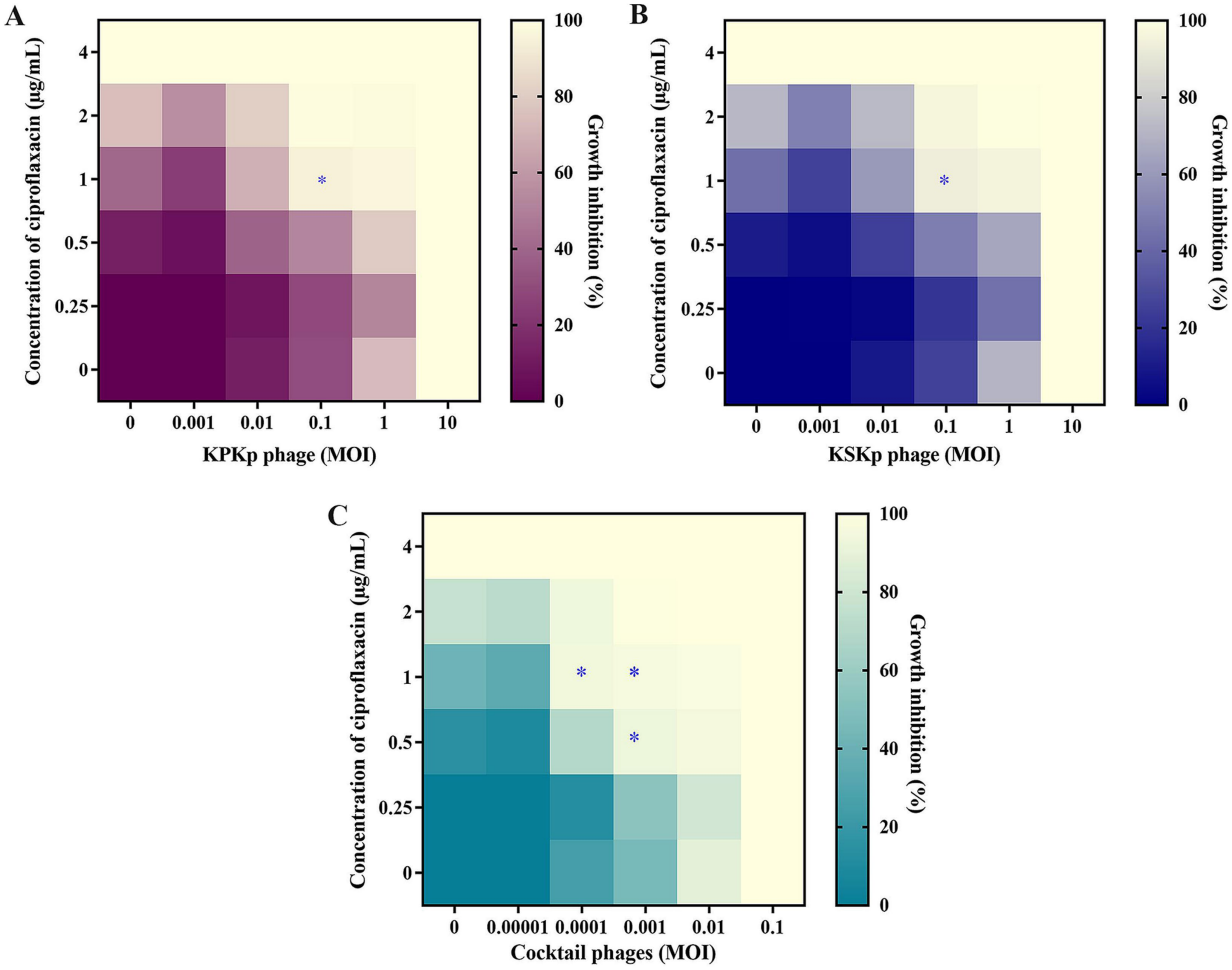
The synergy between CIP and phages in *in vitro*. **(A)** KPKp (MOI 10 to 0.001, rows), **(B)** KSKp (MOI 10 to 0.001, rows), and **(C)** cocktail phages (KPKp and KSKp, rows) (MOI 0.1 to 0.00001). Each phage **(A–C)** showed synergy with CIP (4 to 0.25 μg/mL, columns); the wells in the lower section (last rows) contained a single treatment of phages, while those in the left section (first column) contained antibiotic treatment. The bottom-left well (first well or 0) served as a no-treatment control. The mean reduction in bacterial density at the endpoint is color-coded by shades of violet (for KPKp), blue (for KSKp) and green (for cocktail phages - KPKp & KSKp). The star (*) indicates bacterial clearance of more than 90% based on synergism.

This study explored the synergistic effects of phages (KPKp, KSKp, and a phage cocktail) combined with CIP against *K. pneumoniae* ATCC 700603 using a checkerboard assay (Figure 3). Ciprofloxacin, a fluoroquinolone antibiotic that inhibits bacterial DNA replication, was selected due to its partial activity against the test strain, as evidenced by its measurable MIC of 4 μg/mL. Among the panel of antibiotics tested (Tables 1, 4), CIP was the only agent that displayed an interpretable MIC, while others showed high-level resistance, rendering them unsuitable for synergy evaluation. This made ciprofloxacin a suitable candidate for assessing PAS through dose-reduction assays. The MIC of CIP for *K. pneumoniae* was determined to be 4 μg/mL. In this context, “sub-lethal” or “sub-inhibitory” doses specifically refer to CIP concentrations of 1 μg/mL and 0.5 μg/mL, representing 4-fold and 8-fold reductions from the MIC, respectively. Lower CIP concentrations were tested in combination with individual phages (KPKp and KSKp) and the phage cocktail to assess their synergistic antibacterial activity.

The results revealed that CIP at 1 μg/mL (a 4-fold reduction from the MIC of 4 μg/mL) combined with individual phages at an MOI of 0.1 reduced bacterial growth by over 90% for both KPKp (Figure 3A) and KSKp (Figure 3B). Notably, the phage cocktail showed even stronger synergy, achieving over 90% inhibition in three combinations: CIP at 1 μg/mL with cocktail phages at MOIs of 0.001 and 0.0001, and CIP at 0.5 μg/mL with cocktail phages at an MOI of 0.001 (Figure 3C).

Most importantly, CIP at 0.5 μg/mL combined with the phage cocktail at an MOI of 0.001 (Figure 3C) demonstrated significant synergistic potential, reducing bacterial growth by more than 95%. These combinations represent an 8-fold reduction in CIP MIC and phage MOI reductions ranging from 100-fold (from 0.1 to 0.001) to 1,000-fold (from 0.1 to 0.0001) (Figure 3C). This underscores the efficacy of PAS therapy at markedly reduced concentrations of both CIP and phages, as depicted in the heatmap (Figure 3C). As a potential strategy for fighting MDR pathogens like *K. pneumoniae*, PAS therapy reduces the danger of resistance development and the adverse effects associated with increasing antibiotic usage by obtaining strong bacterial suppression with lower doses of antibiotics and phages.

Both KPKp and KSKp showed >90% growth inhibition with CIP (1 μg/mL), with KPKp displaying slightly stronger synergy, likely due to greater stress tolerance. The cocktail outperformed individual phages, indicating complementary activity and enhanced efficacy at lower concentrations.

#### 3.3.2 Evaluation of *Klebsiella pneumoniae* planktonic cell vulnerability

##### 3.3.2.1 Comparison of individual phages and cocktail treatment

Spectroscopic analysis revealed that individual phages KPKp and KSKp suppressed bacterial growth by ~96% (Supplementary Figure 5A) and ~94% (Supplementary Figure 5B), respectively, over 24 h. In contrast, the phage cocktail exhibited significantly greater efficacy, achieving ~99% suppression within 9 h, corroborating the CFU data.

**Figure 4:**
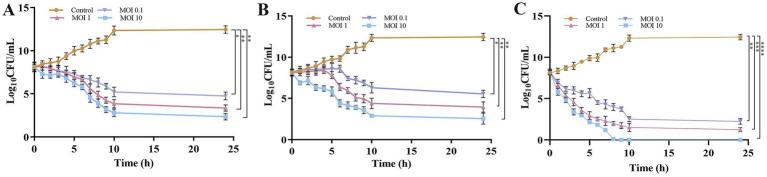
Evaluation of phage therapeutic activity against *K. pneumoniae* planktonic cells through time-kill kinetics (CFU). Bacterial counts (CFU/mL) were measured following treatment with **(A)** KPKp, **(B)** KSKp, and **(C)** the phage cocktail (KPKp and KSKp) against *K. pneumoniae* ATCC 700603. The symbols *, **, ***, and **** indicate statistical significance for *p* < 0.05, *p* < 0.01, *p* < 0.001, and *p* < 0.0001. Standard deviations are shown by error bars. All experiments were conducted in triplicate, both experimentally and biologically.

CFU enumeration further demonstrated that the phage cocktail (KPKp and KSKp) was significantly more effective in lysing planktonic *K. pneumoniae* at MOI 10 than individual phages. KPKp and KSKp achieved bacterial reductions of ~10.1 log (Figure 4A) and ~9.9 log (Figure 4B), respectively, at 24 h. In contrast, the phage cocktail resulted in a significantly greater reduction of ~12.4 log within 9 h (Figure 4C, *p* < 0.0001), highlighting its superior efficacy and rapid bactericidal activity.

The observed rapid bacterial lysis and substantial pathogenic load reduction underscore the therapeutic potential of this phage cocktail for future clinical applications. KPKp and KSKp both exhibited strong bactericidal activity, with KPKp showing slightly higher suppression in spectrophotometric and CFU assays, suggesting greater lytic potency. These differences may reflect variations in adsorption rate, burst size, or host range. The cocktail demonstrated enhanced efficacy, highlighting the advantage of phage combination therapy.

##### 3.3.2.2 Evaluation of synergistic PAS treatment

**Figure 5:**
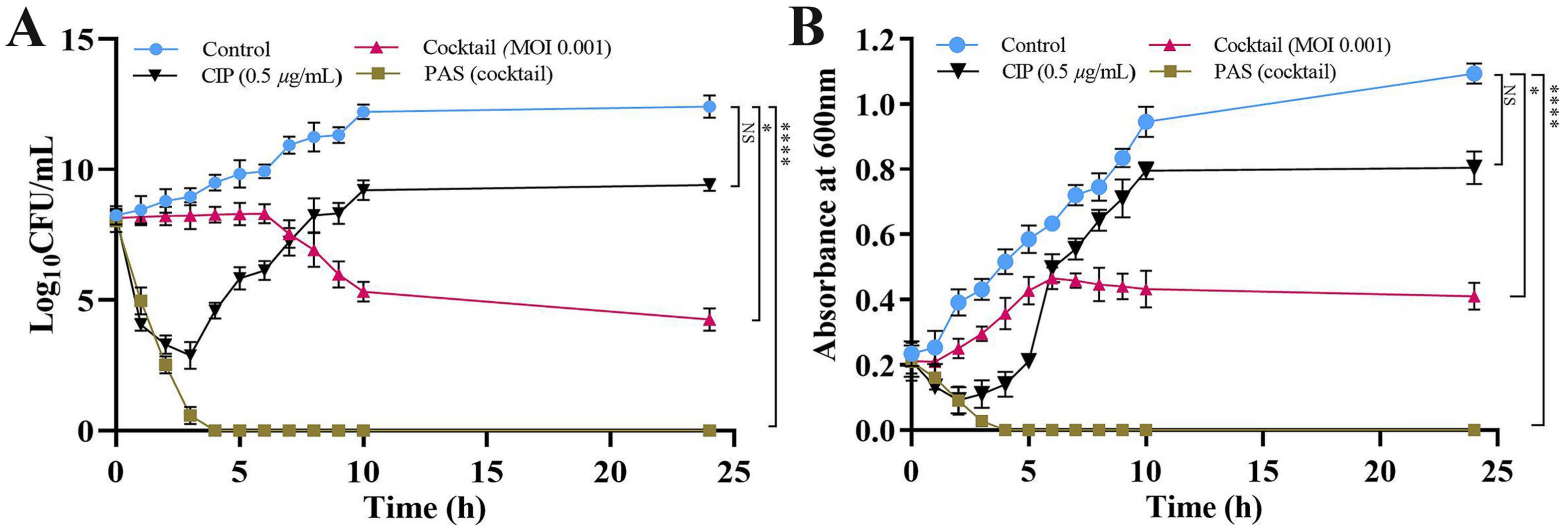
PAS treatment of *K. pneumoniae* planktonic cells is used to measure phage activity. **(A)** CFU/mL counts and **(B)** optical density (OD) at 600 nm. The PAS treatment was applied to *K. pneumoniae* ATCC 700603 planktonic cells. Asterisks *, **, ***, and **** indicate statistical significance at *p* < 0.05, 0.01, 0.001, and 0.0001, respectively. Error bars represent standard deviations. All experiments were performed in biological and technical triplicates.

The PAS treatment, which combines a phage cocktail (MOI 0.001) with CIP at 0.5 μg/mL, demonstrated remarkable efficacy in reducing bacterial load with a lower phage concentration compared to individual treatments. When administered independently, the single phages (KPKp or KSKp) or the phage cocktail (KPKp and KSKp) showed limited effectiveness. Specifically, at MOI 0.001, the phage cocktail alone resulted in a modest ~8.1-log reduction, whereas CIP alone achieved only a ~ 3-log reduction and failed to significantly suppress bacterial growth over 24 h. In contrast, the PAS treatment achieved a ~ 8.3-log reduction within just 3 h, comparable to the cocktail-alone treatment at a higher MOI but achieved in a much shorter timeframe (Figure 5A).

Spectroscopic analysis further corroborated these findings, revealing significantly greater bacterial growth suppression with PAS treatment (*p* < 0.0001) compared to either the phage cocktail or CIP alone. At 24 h, the phage cocktail alone at MOI 0.001 achieved ~62% bacterial reduction, while CIP alone resulted in ~26% reduction. Remarkably, the PAS treatment (phage cocktail at MOI 0.001 combined with CIP) achieved ~99% bacterial reduction within just 3 h (Figure 5B).

These findings indicate a potential synergistic interaction between the phage cocktail and CIP under the tested conditions, resulting in enhanced bacterial clearance. Moreover, the reduction in the required phage concentration may provide practical advantages, including lower production costs and decreased exposure to high phage doses. However, the observed synergy is likely influenced by specific experimental parameters, and further investigations in clinically relevant models are required to determine its broader therapeutic applicability.

Although KPKp and KSKp exhibited limited individual efficacy at an MOI of 0.001, the enhanced antibacterial activity observed in the PAS treatment suggests a synergistic interaction not only between phages and antibiotics but also between the two phages. The potent lytic activity of KPKp may play a primary role in initial bacterial clearance, while KSKp potentially contributes by targeting resistant subpopulations or mitigating the emergence of resistance.

#### 3.3.3 Evaluation of biofilm suppression by single phages, phage cocktail, and PAS

This assay evaluates the effectiveness of individual phages, cocktail phages and PAS in suppressing *K. pneumoniae* biofilms. The goal is to identify effective strategies to enhance phage proliferation and reduce biofilm density, improving treatment outcomes for chronic infections.

**Figure 6:**
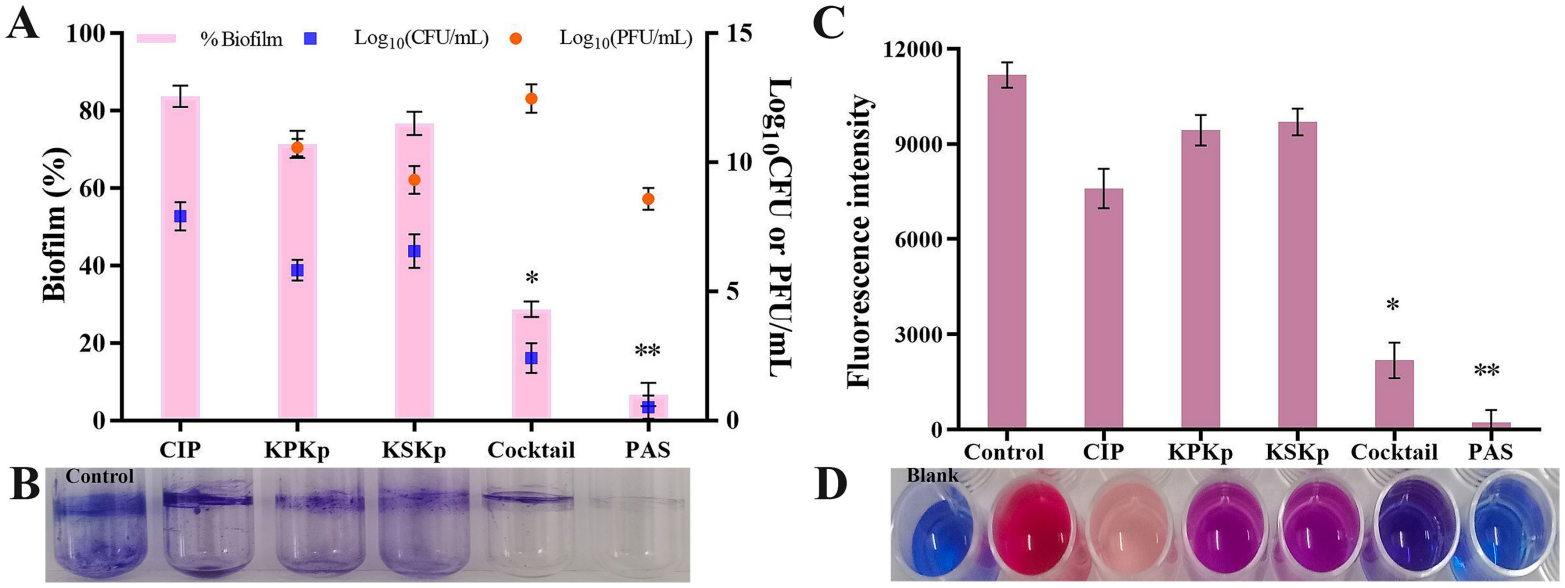
Therapeutic effect on biofilms of *K. pneumoniae* ATCC 700603. **(A)** CIP (MIC 4 μg/mL), KPKp (MOI 10), KSKp (MOI 10), cocktail phages (MOI 10), and PAS (cocktail phages at MOI 0.001 + CIP 0.5 μg/mL) were used to treat inhibitory potential *K. pneumoniae* biofilms. **(B)** Biofilms are stained with 1% CV, and the total biomass of the biofilm is measured at OD_570_. **(C,D)** The above **(A)** treatments were further validated by Alamar Blue assay. The pink color indicates metabolically active cells. The blue color indicates no metabolic activity. *, ** indicate significant values at *p <* 0.05, *p <* 0.01, respectively. Error bars represent standard deviations. Each experiment was conducted three times.

The obtained results show that the density of *K. pneumoniae* biofilms was effectively reduced by phages (individual and cocktail) at MOI 10 and PAS (CIP 0.5 μg/mL and cocktail phages MOI 0.001) (Figures 6A,B). After 24 h, the most substantial reduction was achieved with ~16.3% (CIP 4 μg/mL), ~28.7% (KPKp), ~23.3% (KSKp), ~71.4% (cocktail phages MOI 10) and ~93.4% (PAS) (*p* < 0.01) (Figures 6A,B).

These findings were confirmed by CFU enumeration on TSA plates (Figure 6A, blue color symbol). Compared to the phage cocktail, PAS treatment significantly reduced bacterial counts (Figure 6A, blue color symbol). Additionally, the number of PFU was determined, showing that the concentration of KPKp and KSKp phages increased by approximately 2.5- and 1.3-log folds, respectively (Figure 6A, orange color symbol). The phage cocktail resulted in an ~4.4-log increase, while the PAS sample exhibited a ~ 3.6-log increase, even at a low MOI (0.001). After 24 h, no biofilm recurrence was observed, highlighting the potent antibiofilm activity of phages against sessile *K. pneumoniae* cells (*p* < 0.01).

The data clearly indicate that PAS, combining low concentrations of CIP and cocktail phages, offers superior biofilm reduction compared to phages or antibiotic alone. PAS not only achieved the greatest reduction in biofilm mass but also enhanced phage proliferation within the biofilm matrix, preventing recurrence of *K. pneumoniae*. These findings suggest that PAS is an effective therapeutic strategy for targeting resilient biofilms, potentially providing a powerful approach for treating chronic *K. pneumoniae* infections.

KPKp consistently showed greater efficacy in biofilm reduction and phage replication (2.5-log fold compared to 1.3-log fold for KSKp), suggesting superior penetration or replication within the biofilm matrix. Despite this, both phages contributed to the cocktail’s enhanced performance, with KPKp likely serving as the primary agent and KSKp complementing by broadening host range and improving stability.

##### 3.3.3.1 Resazurin-based metabolic activity assay

Biofilm cells in the control samples showed evidence of metabolically active cells by remaining pink for 24 h (Figures 6C,D). Biofilm cells treated with CIP (light pink), KPKp (violet) and KSKp (violet) showed metabolically active cells. But cocktail phages (KPKp and KSKp) and PAS were only in blue color represent metabolically active cells were significantly reduced. This demonstrates the effectiveness of both phage therapy and PAS in disrupting the metabolic function of *K. pneumoniae* biofilms.

##### 3.3.3.2 Microscopic examination

**Figure 7:**
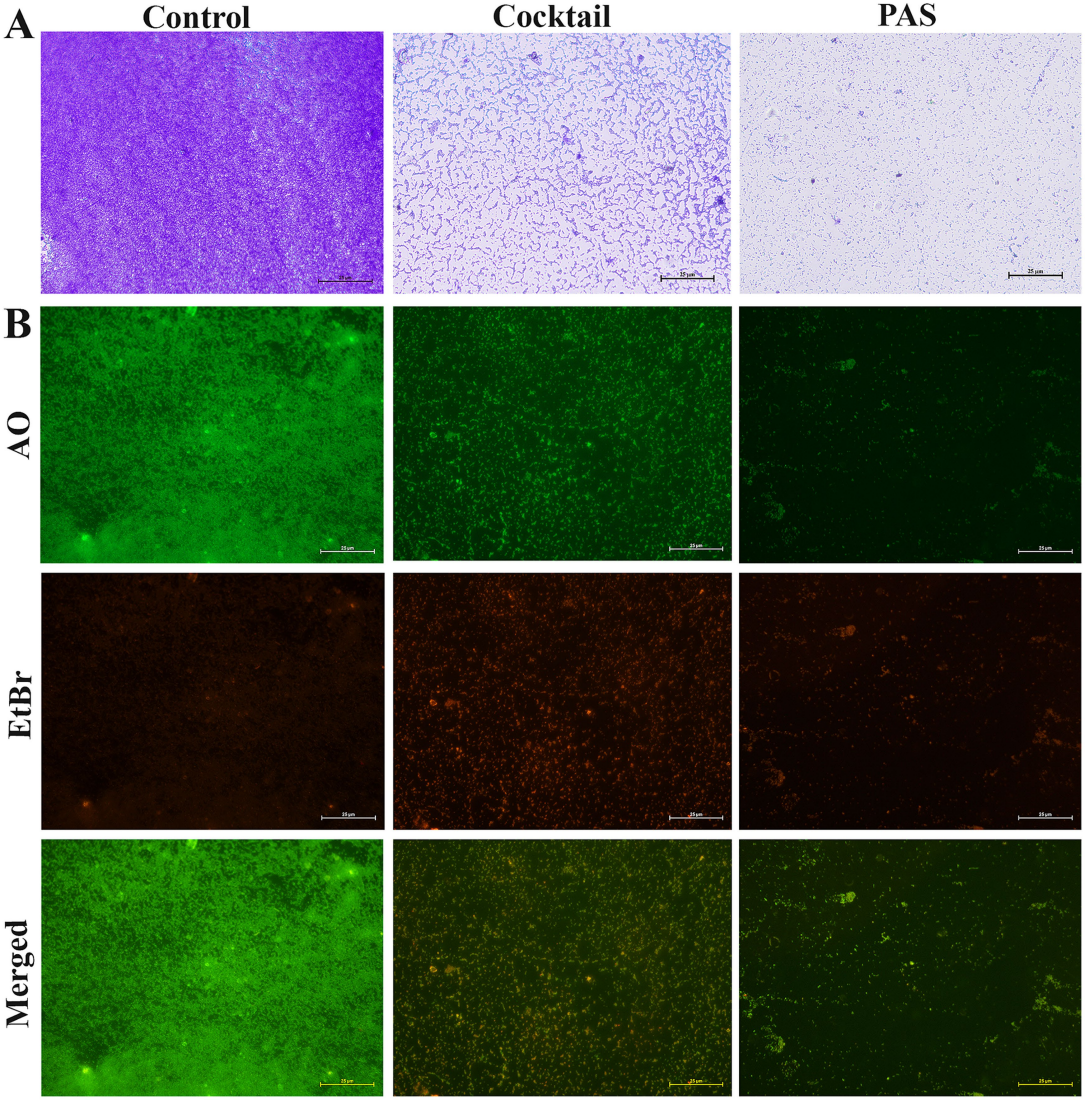
Micrographs of phage infectivity on *K. pneumoniae* ATCC 700603 biofilms. The images illustrate the infectivity of cocktail phages (KPKp & KSKp) and PAS on *K. pneumoniae* (ATCC 700603) biofilms. Without any treatment called control. **(A)** Light microscopy images comparing biofilm architecture before and after treatment with phages. (scale bar = 25 μm). **(B)** Fluorescence microscopy results showing live (green) and dead (red) cells in biofilms stained with AO and EtBr, illustrating the effect of phage treatment on biofilm viability (scale bar = 25 μm).

**Figure 8:**
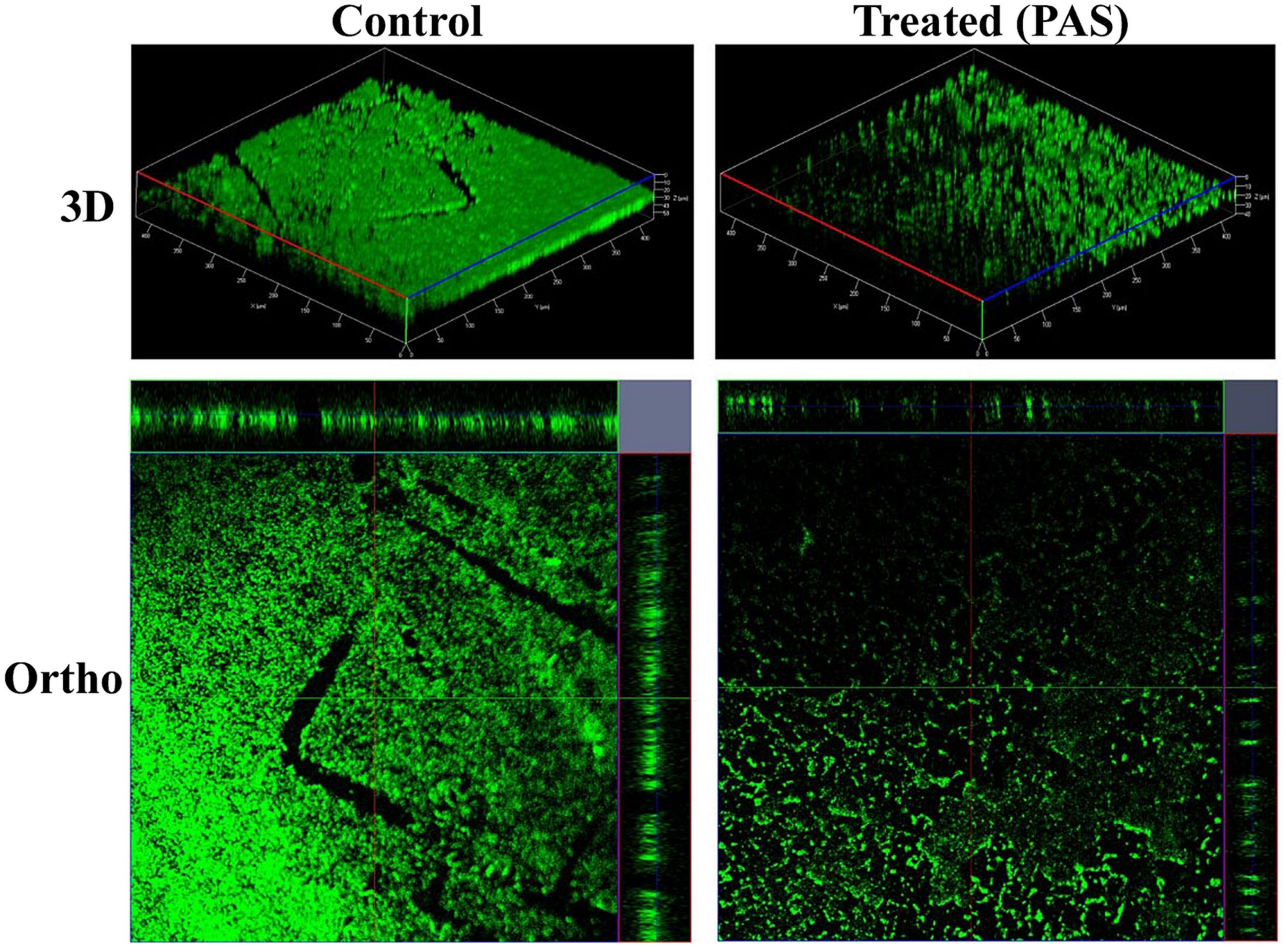
CLSM 3D and orthographic images showing the developmental phases of *K. pneumoniae* biofilms.

To assess the impact of cocktail phages and PAS on *K. pneumoniae* biofilms, a combination of light microscopy and fluorescence microscopy (Figure 7), along with CLSM (Figure 8), was employed. The use of multiple imaging techniques was critical to achieve a comprehensive evaluation of both the structural and functional changes in the biofilm, which could not be accomplished by a single method alone.

Light microscopy provided an initial overview of biofilm architecture and matrix entanglement. In control samples, densely packed biofilm layers embedded in extracellular polymeric substances were observed (Figure 7A). In contrast, cocktail phage-treated samples showed partially disrupted cellular organization, while PAS-treated samples exhibited a more pronounced dispersion, indicating greater biofilm degradation.

Fluorescence microscopy with AO and EtBr staining assessed bacterial viability (Figure 7B). EtBr selectively stained non-viable cells (red), while AO stained both viable (green) and non-viable cells (red). This technique enabled viability-based differentiation not possible with light microscopy. Phage cocktail and PAS-treated samples exhibited intense red fluorescence, indicating extensive cell death, while control samples showed green fluorescence, indicating viable cells.

CLSM provided high-resolution 3D and orthographic views of the biofilm structure. Control samples exhibited thick, uniform biofilms with strong green fluorescence, suggesting high biomass and metabolic activity. In contrast, PAS-treated samples showed disrupted architecture, reduced fluorescence intensity, and decreased thickness, indicating diminished cell density and compromised biofilm integrity.

The integration of these three imaging techniques light microscopy for structural overview, fluorescence microscopy for viability assessment, and CLSM for high-resolution spatial visualization offered a multi-dimensional understanding of biofilm disruption. This complementary approach enabled a more robust interpretation of the efficacy of phage and PAS treatments than any single method could provide.

Thus, employing all three methods was necessary to capture the complex effects of therapeutic interventions on biofilm structure and viability, supporting the potential of phage-based strategies in combating *K. pneumoniae* biofilm-associated infections.

### 3.4 Evaluation of effectiveness *in vivo*

#### 3.4.1 Analyzing *Galleria mellonella* larvae for pathogenicity and phage treatment

**Figure 9:**
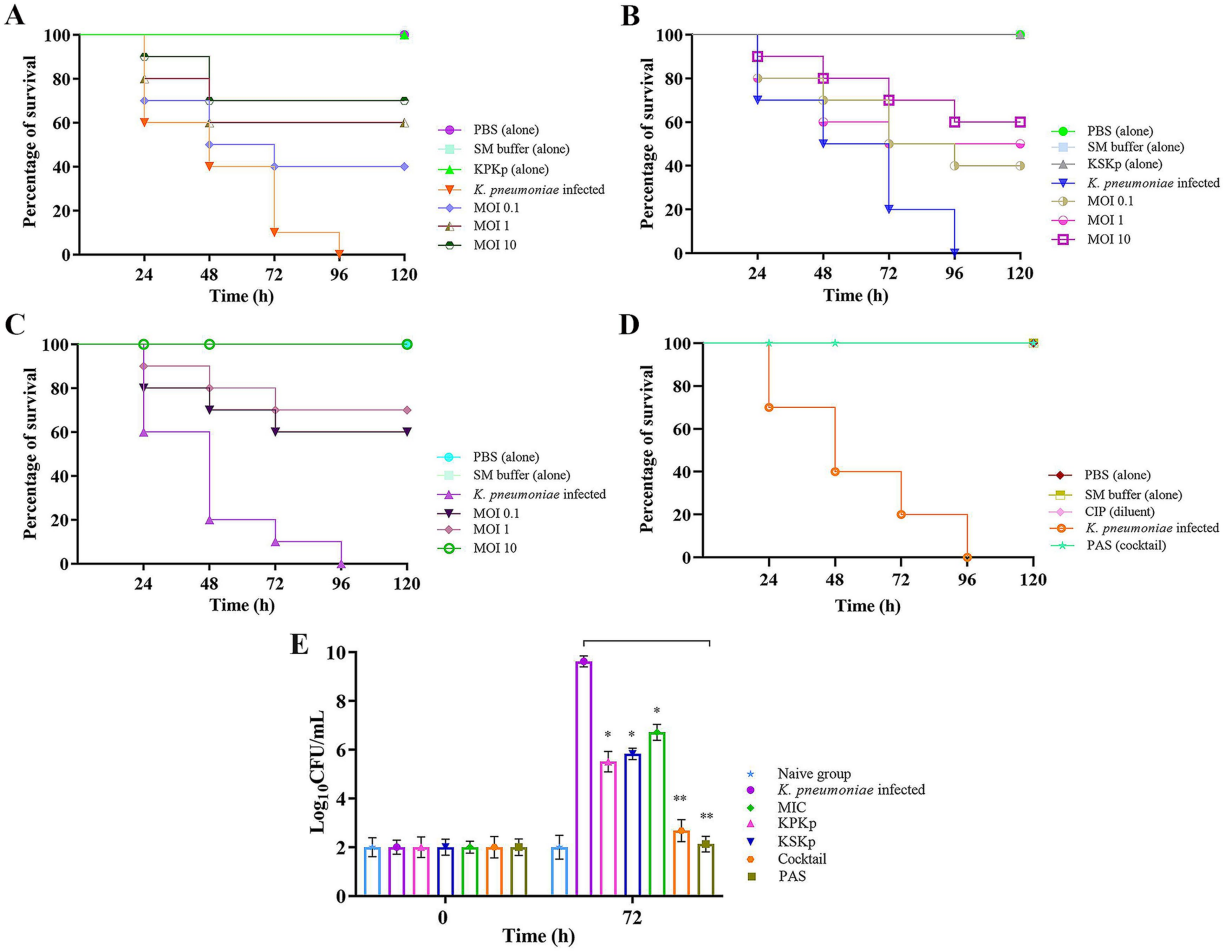
Survival analysis of *G. mellonella* larvae infected with *K. pneumoniae*. Kaplan–Meier survival curve showing the percentage of surviving *G. mellonella* larvae treated with individual phages, phage cocktail, and PAS. **(A)** KPKp at different MOIs (0.1, (1), and 10). **(B)** KSKp at different MOIs (0.1, 1, and 10). **(C)** Phage cocktail at different MOIs (0.1, 1, and 10). **(D)** PAS treatment (cocktail phages at MOI 0.001 and CIP at 0.5 μg/mL) administered simultaneously with infection. **(E)** Assessment of bacterial burden in larvae; simultaneous treatment with individual phages, phage cocktail, antibiotic alone, and PAS resulted in a marked reduction in CFU counts, indicating effective control of bacterial load and enhanced survival. Statistical significance was determined using log-rank tests for survival analysis and unpaired t-tests for CFU counts, with significance levels indicated as *p* < 0.001 and *p* < 0.0001.

The pathogenicity of *K. pneumoniae* in *G. mellonella* larvae was evaluated to determine melanization and survival following bacterial infection. At a dose of 10 μL per larvae, comprising approximately 1 × 10^8^ CFU/mL, *K. pneumoniae* was injected into the larvae. The larvae showed signs of gradually becoming melanized. The mortality rate of *K. pneumoniae*-infected larvae increased in a dose-dependent manner by day 5, with almost half of the larvae dying within two days [Figures 9A–D; Supplementary Figure 6 (*K. pneumoniae* infected group)]. In contrast, larvae in the uninfected control groups [PBS, SM buffer, CIP diluent (d. water with a small drop of HCl)], showed no signs of melanization and exhibited no mortality [Figures 9A–D; Supplementary Figure 6 (control groups)].

In addition, some larvae were injected with phage alone (KPKp and KSKp) without bacterial infection to assess potential toxicity or infectivity (Figures 9A,B; Supplementary Figure 6). Throughout the observation period, live larvae successfully harbored phages without showing any signs of mortality or melanization. In contrast, larvae infected solely with *K. pneumoniae* exhibited melanization and ~90% mortality within 72 h compared to the control groups [Figures 9A,B; Supplementary Figure 6 (Treatment groups-KPKp &KSKp)].

Larvae were given a single dose of phages with *K. pneumoniae* infection to evaluate the efficacy of simultaneous therapeutic treatment. This approach demonstrated a significant effect in controlling the pathogen. Larvae treated with KPKp [Figure 9A; Supplementary Figure 6 (KPKp alone)] and KSKp [Figure 9B; Supplementary Figure 6 (KSKp alone)] phages at MOI 10 showed survival rates of approximately ~70 & 60%, respectively. In the cocktail group [Figure 9C; Supplementary Figure 6 (cocktail)], survival rates increased with phage concentration, achieving ~60, 70, and 100% survival at MOI levels of 0.1, 1 and 10, respectively. Additionally, a combination of cocktail phages (MOI 0.001) and CIP (0.5 μg/mL) resulted in 100% survival (Figure 9D; Supplementary Figure 6).

Bacterial burden analysis (Figure 9E) in *G. mellonella* larvae demonstrated significant reductions in bacterial load following treatment with KPKp (~42.73%) and KSKp (~39.39%). Under MIC-treated conditions, bacterial load was reduced by ~30.24%. In comparison, treatment with the phage cocktail and PAS resulted in even greater reductions of ~72.13% and ~77.91%, respectively (*p* < 0.001). These findings highlight the enhanced therapeutic efficacy of phage therapy and PAS in combating *K. pneumoniae* infection.

#### 3.4.2 Deciphering tissue histology

**Figure 10:**
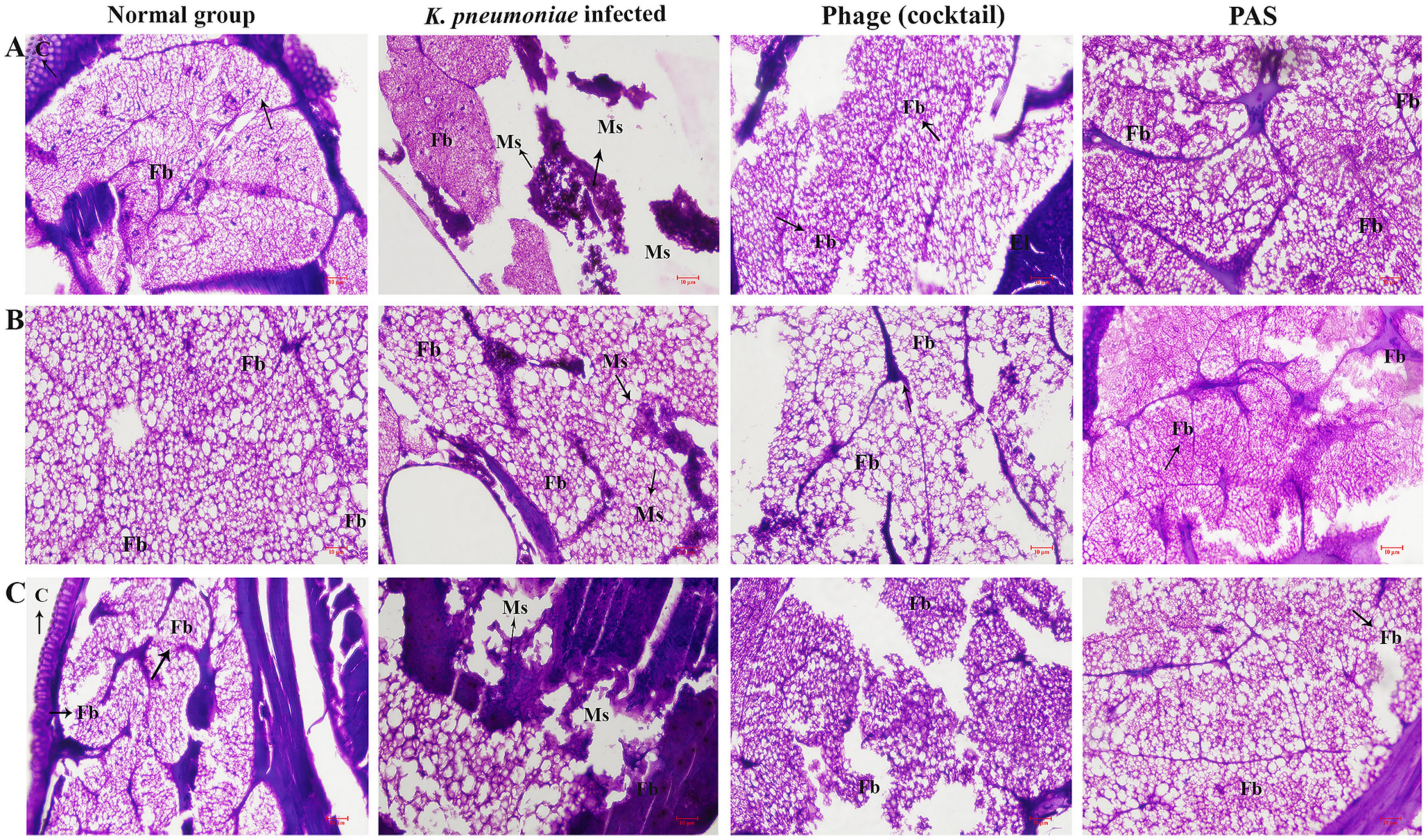
Histopathological analysis of larvae tissues post-treatment. Microscopic examination reveals tissue damage in infected larvae compared to controls. Histological sections from naive control larvae show intact tissues with no abnormalities. Tissues from larvae infected with *K. pneumoniae* display extensive damage, characterized by melanization and disruption of cellular architecture. Larvae treated with cocktail show improved tissue integrity with minimal damage. The therapeutic potential of PAS and phage therapy in reducing *K. pneumoniae*’s virulence in the *G. mellonella* model is highlighted by this investigation. Different portions of the same larvae’s microscopic picture are represented by panels **(A–C)**. C-cuticle, El-epithelial layer, Ms-melanized structure, Fb-fat body, and h-hematoxylin, Mf-muscular fiber.

Additionally, histopathological examination provided insights into the antivirulence efficacy at the tissue level. At 120 h post-inoculation, one animal from each group underwent histopathological analysis (Figure 10). Microscopic evaluation of tissue smears from the naive control group showed no abnormalities. In contrast, tissue damage and melanised structure was evident in the *K. pneumoniae* infected control group, whereas the cocktail phages and PAS treatment groups displayed healthy tissues.

The histopathological examination of *G. mellonella* larvae demonstrated that phage cocktail and PAS treatments effectively preserved tissue structure, reducing the signs of melanization and damage observed in the infected control group. This suggests that combination therapy significantly improves tissue health in infected larvae, providing further evidence of the therapeutic potential of PAS *in vivo*.

## 4 Discussion

MDR bacterial infections present significant clinical challenges, highlighting the urgent need for novel antimicrobial strategies. Phage therapy offers a promising alternative or adjunct to antibiotics, with high specificity for antibiotic-resistant pathogens like *K. pneumoniae* (Terreni et al., 2021). This study investigated the therapeutic potential of two phages, KPKp and KSKp, individually and in combination with CIP against MDR *K. pneumoniae*.

Phage cocktails are developed to overcome the limitations of monophage therapies, such as the emergence of resistant mutants and narrow host range (Yuan et al., 2019). The use of multiple phages increases the spectrum of bacterial targets and decreases the likelihood of bacterial resistance (Oechslin, 2018). Our results show that the phage cocktail provided enhanced therapeutic efficacy compared to individual phages, aligning with the principle that combining different phages broadens lytic activity and mitigates the development of resistance. The combination of phages with antibiotics, particularly the PAS strategy, has been recognized as a powerful therapeutic approach, offering complementary mechanisms of action. Phages typically lyse bacteria by targeting specific surface receptors, while CIP inhibits bacterial DNA replication by targeting DNA gyrase (Zhao et al., 2024; Rafiei and Bouzari, 2024). This dual-targeting approach significantly enhances bacterial clearance and reduces the emergence of resistance compared to monotherapy with either agent alone.

The synergistic efficacy of PAS was evident in both planktonic and biofilm cultures. CIP, known for its ability to penetrate biofilm matrices, facilitates phage access to bacteria embedded in the biofilm, thereby enhancing phage proliferation and bacterial lysis (Holger et al., 2023). Additionally, sub-inhibitory concentrations of antibiotics, such as CIP, can induce bacterial stress responses, such as filamentation, which may further sensitize bacteria to phage infection (Ronayne et al., 2016). Sub-lethal concentrations of CIP were defined as those below the MIC of 4 μg/mL, specifically 1 μg/mL and 0.5 μg/mL, which demonstrated partial inhibition when used alone and showed pronounced synergistic effects when combined with phages. The use of sub-lethal doses in PAS therapy highlights the potential to reduce antibiotic usage while enhancing antibacterial efficacy, mitigating both toxicity and resistance development risks. Notably, combining CIP at 0.5 μg/mL with a low phage MOI (0.001) resulted in over 99% reduction of planktonic cells within 3 h, surpassing the efficacy of monotherapies. These findings are consistent with previous studies showing that low antibiotic concentrations enhance phage penetration and replication within biofilms, as well as reduce host cytotoxicity (Ali et al., 2023; Fungo et al., 2023).

Various antibiotics were initially tested, including carbapenems (meropenem, imipenem), cephalosporins (cefepime, ceftriaxone, ceftazidime), monobactams (aztreonam), fluoroquinolones (CIP, levofloxacin), and aminoglycosides (gentamicin). Due to high resistance profiles, CIP was chosen for further PAS evaluation, showing measurable MIC (4 μg/mL) and compatibility with phage therapy. When combined with phages, CIP achieved substantial reductions in bacterial growth at concentrations well below its MIC, demonstrating the clinical potential of PAS to reduce antibiotic dosages and minimize associated toxicities (Foyle et al., 2023; Sharma et al., 2023).

PAS demonstrated enhanced antibacterial activity against *K. pneumoniae* with CIP at sub-inhibitory concentrations (0.5 and 1 μg/mL), corresponding to 8-fold and 4-fold reductions from the MIC (4 μg/mL). In contrast, CIP monotherapy at or below the MIC lacks efficacy and may promote resistance. Sub-inhibitory CIP exposure failed to control infection and selected for quinolone-resistant strains in an *in vivo G. mellonella* model (Panis et al., 2024). Additionally, transcriptional profiling of *K. pneumoniae* under CIP stress revealed activation of the SOS response and efflux pump systems, indicating the induction of stress-mediated resistance pathways at MIC levels (Alvi et al., 2021). By achieving bacterial clearance at sub-MIC concentrations, PAS minimizes the selective pressure typically associated with higher antibiotic doses, thereby limiting the activation of resistance mechanisms.

Beyond pharmacological synergy, molecular and physiological differences between the two phages suggest complementary therapeutic roles. Phages from the *Ackermannviridae* family, such as KPKp, are characterized by larger genomes that encode a broad array of replication-related genes and structural proteins, providing greater adaptability to diverse bacterial conditions (Hendrix, 2009; Hatfull, 2018; Yuan and Gao, 2017). These phages often carry long tail fiber proteins and multiple tRNAs, facilitating broader host interactions and efficient replication (Hatfull, 2018). Conversely, phages in the *Straboviridae* family, like KSKp, are efficient at adsorption, possess short latent periods, and exhibit high burst sizes, all of which contribute to rapid bacterial clearance (Adriaenssens et al., 2020; Mirzaei and Maurice, 2017). These attributes make them particularly suitable for acute infection control.

The observed differences between KPKp and KSKp support the rationale for combining them in a cocktail formulation. Phages with complementary kinetics and host range profiles are more likely to enhance treatment efficacy and minimize resistance development (Forti et al., 2018). While KPKp produced plaques with surrounding halos indicative of depolymerase activity, which aids in biofilm degradation, KSKp lacked this trait. Phages encoding depolymerases have been shown to enhance biofilm penetration and disruption (Latka et al., 2021), making these structural and functional differences important for their combined use in a phage cocktail.

Genomic analyses revealed sequence divergence between the isolated phages and previously described *Klebsiella*-infecting phages. One phage displayed high sequence identity with a known *Klebsiella* phage, while the other exhibited greater genetic variation. This genomic diversity reflects the adaptability of phages to different bacterial receptors and ecological niches, a phenomenon previously reported in other studies (Adriaenssens et al., 2020; Ferriol-González and Domingo-Calap, 2024). Phylogenetic clustering and comparative genome alignments supported their classification within established *Klebsiella*-targeting phage groups, aligning with prior taxonomic frameworks (Hennart et al., 2022).

The present study evaluates the therapeutic efficacy of two lytic *Klebsiella*-targeting phages, KPKp and KSKp, highlighting the superior potential of KPKp as a next-generation therapeutic agent. Whole genome analysis confirmed the strictly lytic nature of both phages, with the absence of lysogeny-associated genes, ensuring their clinical safety. Notably, KPKp, classified as a jumbo phage, possesses an expansive genome encoding a suite of autonomous replication and transcriptional machinery, including consecutively organized DNA helicases (ORFs 28–33), ligases (ORFs 34–37), polymerases (ORFs 38–41), primases (ORFs 42–43), and topoisomerase (ORF 44). This structured genomic architecture allows KPKp to replicate independently of host cellular systems, thereby conferring a higher burst size, broad host range as validated by EOP & One-step growth curve analyses Although KSKp, with a genome size of 167,101 bp, does not meet the classification threshold for jumbo phages, its genome exhibits distinct replication features, including DNA helicases (ORFs 55–56) and a DNA primase (ORF 217), supporting a highly productive lytic cycle. The presence of DNA helicases in ORF 55 & 56 and DNA primase in ORF 217 aids KSKp in maintaining its high burst size.

WGS and annotation studies confirmed the strictly lytic lifestyle of both phages, evidenced by the complete absence of lysogeny-associated genes, including integrase, excisionase and repressor elements, thereby eliminating the risk of prophage integration or horizontal gene transfer an essential criterion for clinical safety. These features coupled with cocktail and PAS systems validated in this study will surpass the functional limitations of conventional phage therapy, enhancing its therapeutic potential in MDR infections of *K. pneumoniae*.

*In vitro*, substantial reductions in *K. pneumoniae* planktonic cell counts were observed following both phage cocktail and PAS treatments, highlighting the potential of phage-antibiotic combinations for enhancing therapeutic efficacy. The phage cocktail alone achieved a ~ 12.4-log reduction in bacterial counts over 9 h, consistent with previous studies demonstrating potent bactericidal activity of phages in planktonic systems (Abedon, 2015). Notably, PAS treatment, even at low CIP and phage dosages, achieved similar reductions within just 3 h, underscoring the synergistic potential of phage-antibiotic combinations. This synergy not only enhances therapeutic outcomes but also significantly reduces the required dosages of both agents, minimizing side effects and resistance development (Jin et al., 2023).

In anti-biofilm assays, PAS treatment reduced biofilm density by ~93.4%, compared to ~71.4% with phage cocktail alone. This enhanced efficacy aligns with previous studies that reported superior biofilm disruption with combined phage-antibiotic treatments, which are particularly important for eradicating biofilms that are resistant to antibiotics alone (Subramanian, 2024). Metabolic activity assays revealed minimal viability in PAS-treated biofilms, supporting its efficacy in targeting both dormant and metabolically active bacteria within biofilms. Microscopic analysis using light microscopy, fluorescence microscopy, and CLSM revealed significant biofilm structural disintegration and a high proportion of dead cells following PAS treatment, supporting previous findings that phages can effectively penetrate and dismantle biofilm matrices (Scherr et al., 2015; Amankwah et al., 2021).

The *G. mellonella* infection model served as an *in vivo* validation of phage safety and efficacy. Infected larvae exhibited ~90% mortality within 72 h, reflecting the virulence of *K. pneumoniae* in this model, which is widely used for preclinical infection studies (Yu et al., 2024). Phage-only treatment improved survival to 60–70%, consistent with other studies showing therapeutic benefits of phage therapy in *G. mellonella* (Kelly and Jameson, 2024). Remarkably, PAS-treated larvae achieved 100% survival and exhibited significant bacterial clearance, further demonstrating the effectiveness of combined phage-antibiotic therapy *in vivo*. Histopathological analysis confirmed preserved tissue structure in PAS-treated larvae, while untreated controls showed extensive melanization, indicative of infection-related tissue damage. These findings align with previous studies showing that PAS offers robust protection in animal models (Quansah et al., 2022).

Phage therapy can modulate host immune responses, with evidence indicating both pro- and anti-inflammatory effects. Van Belleghem et al. (2017) reported that exposure of peripheral blood mononuclear cells to phages targeting *Staphylococcus aureus* and *Pseudomonas aeruginosa* induced cytokines such as IL-1*α*, IL-6, TNF-α, and IL-10, suggesting a complex immunomodulatory profile. Additionally, Roach et al. (2017) demonstrated that neutrophil activity was essential for effective phage-mediated clearance of *P. aeruginosa* in a murine pneumonia model, emphasizing the concept of immunophage synergy. These findings highlight the importance of evaluating innate immune parameters, including macrophage and neutrophil phagocytosis, to better understand the therapeutic landscape and limitations of phage-based interventions.

The observed PAS effect in this study demonstrated superior antibacterial efficacy compared to phage or antibiotic monotherapy, highlighting its therapeutic potential against MDR *K. pneumoniae*. Similar results have been reported in previous studies. Gorodnichev et al. (2025) showed that specific phage-antibiotic combinations significantly reduced *K. pneumoniae* viability, with the choice of experimental methodology influencing synergy outcomes. De Soir et al. (2024) demonstrated that PAS effectively disrupted *P. aeruginosa* PAO1 biofilms, underlining the potential of PAS in treating biofilm-related infections. These findings support the potential of PAS as a viable strategy against AMR pathogens. In summary, this study evaluates the therapeutic efficacy of lytic *Klebsiella*-targeting phages, KPKp and KSKp, highlighting the superior potential of combining phages with antibiotics like CIP.

## 5 Conclusion

In conclusion, the combined application of PAS combinations offers a promising strategy for tackling the emergence of MDR *K. pneumoniae* strains. This study highlights the efficacy of the lytic phages KPKp and KSKp, both individually and as a cocktail, in significantly reducing bacterial counts and biofilm biomass. The observed synergistic effects when phages are used in conjunction with antibiotics underscore their potential for enhancing therapeutic outcomes against MDR strains. These findings emphasize the need to further explore PAS and to select effective phages as viable alternatives in the fight against antimicrobial resistance.

## Data Availability

The datasets presented in this study can be found in online repositories. The names of the repository/repositories and accession number(s) can be found in the article/Supplementary material.
