# Supplementary material for: Phage (cocktail)-antibiotic synergism: a new frontier in addressing Klebsiella pneumoniae resistance
Source: Front Microbiol. 2025 May 7;16:1588472. doi: 10.3389/fmicb.2025.1588472 (PMC12092377; doi:10.3389/fmicb.2025.1588472)
Supplement: Supplementary file 2 [file Table_2.doc]

**SUPPLEMENTARY FIGURE LEGENDS**

**Supp. Fig. 1. Spot test analysis of phages on bacterial hosts and clinical isolates of *K. pneumoniae*. A)** Spot test analysis of cocktail phages (KPKp and KSKp) on bacterial hosts from different genera, as detailed in Table 2. **B-D)** Spot test assays of various clinical isolates of *K. pneumoniae*, as described in Table 3: B) KPKp phage C) KSKp phage D) Cocktail phages (KPKp and KSKp).

**Supp. Fig. 2. Comparative genomic analysis of KPKp and KSKp phages.**

**A)** Alignment coverage of KPKp against various *Klebsiella* phages, highlighting the regions of homology across other *K. pneumoniae* phage genomes, indicating conserved sequences crucial for phage function. **B)** Alignment coverage of KSKp, illustrating similar conserved regions between other *K. pneumoniae* phages suggesting functional importance in its interaction with host bacteria. **C)** Percentage identity of KPKp with *Klebsiella* phages vB_KpnM_FRZ284, KpnM6E1, JY1, Mineola, and KP13MC5-5 (accession numbers MZ602148.1, MT580897.1, OP734280.1, NC_055748.1, and OP617748.1), demonstrating a high degree of genetic similarity and supporting KPKp's classification within the *Ackermannviridae* family. **D)** Percentage identity of KSKp with *Klebsiella* phages JD18, vB_KpnM_BovinicusUrsus, vB_KpnM_FRZ284, KP1079, and KMI13 (accession numbers KT239446.1, MW021752.1, MZ602148.1, OM630219.1, and MN101229.1), highlighting significant genetic relationships and potential therapeutic relevance in combating MDR *K. pneumoniae*.

**Supp. Fig. 3. Evolutionary link between KPKp and *Klebsiella* phages.** Comparative genome alignment demonstrating the evolutionary relatedness of KPKp with *Klebsiella* phages vB_KpnM_FRZ284, KpnM6E1, JY1, Mineola, and KP13MC5-5 (accession numbers MZ602148.1, MT580897.1, OP734280.1, NC_055748.1, and OP617748.1). The alignment shows high sequence conservation, supporting the classification of KPKp within the *Ackermannviridae* family.

**Supp. Fig. 4. Evolutionary link between KSKp and *Klebsiella* phages.**

Comparative genome alignment illustrating the genetic relatedness of KSKp with *Klebsiella* phages JD18, vB_KpnM_BovinicusUrsus, vB_KpnM_FRZ284, KP1079, and KMI13 (accession numbers KT239446.1, MW021752.1, MZ602148.1, OM630219.1, and MN101229.1). The alignment reveals significant sequence homology and supports the evolutionary connection of KSKp with other virulent phages within the *Straboviridae* family, suggesting its potential therapeutic applications.

**Supp. Fig. 5. Evaluation of phage therapeutic activity against *K. pneumoniae* planktonic cells by OD_600nm_ measurement.** **A**) KPKp, **B**) KSKp, and **C**) the phage cocktail (KPKp and KSKp) activity against *K. pneumoniae* ATCC 700603 was assessed spectrophotometrically at OD_600nm_. Statistical significance is indicated as *, **, ***, and **** for *P*<0.05, *P*<0.01, *P*<0.001, and *P*<0.0001, respectively. Standard deviations are displayed by error bars. Both biological and experimental triplicates were conducted for every experiment.

**Supp. Fig. 6. Morphological observations of *G. mellonella* larvae.**

Photographs depict the health status of larvae at 72 h. Healthy larvae, appearing white-creamy, are shown alongside infected larvae that exhibit signs of melanization and lethargy. Larvae treated with KPKp, showing healthier appearance compared to controls. Larvae treated with KSKp, highlighting improved viability. Cocktail phage treatment, demonstrating enhanced survival. PAS-treated larvae, exhibiting significant resilience against infection. Morphological changes serve as indicators of the effectiveness of various treatments against *K. pneumoniae* infection.

**SUPPLEMENTARY TABLE LEGEND**

**Supp. Table S1**. Identification of ORF and tRNA coding sequences of KPKp and KSKp phages through genome annotation.
