# Supplementary figures and images for: Phage (cocktail)-antibiotic synergism: a new frontier in addressing Klebsiella pneumoniae resistance
Source: Front Microbiol. 2025 May 7;16:1588472. doi: 10.3389/fmicb.2025.1588472 (PMC12092377; doi:10.3389/fmicb.2025.1588472)

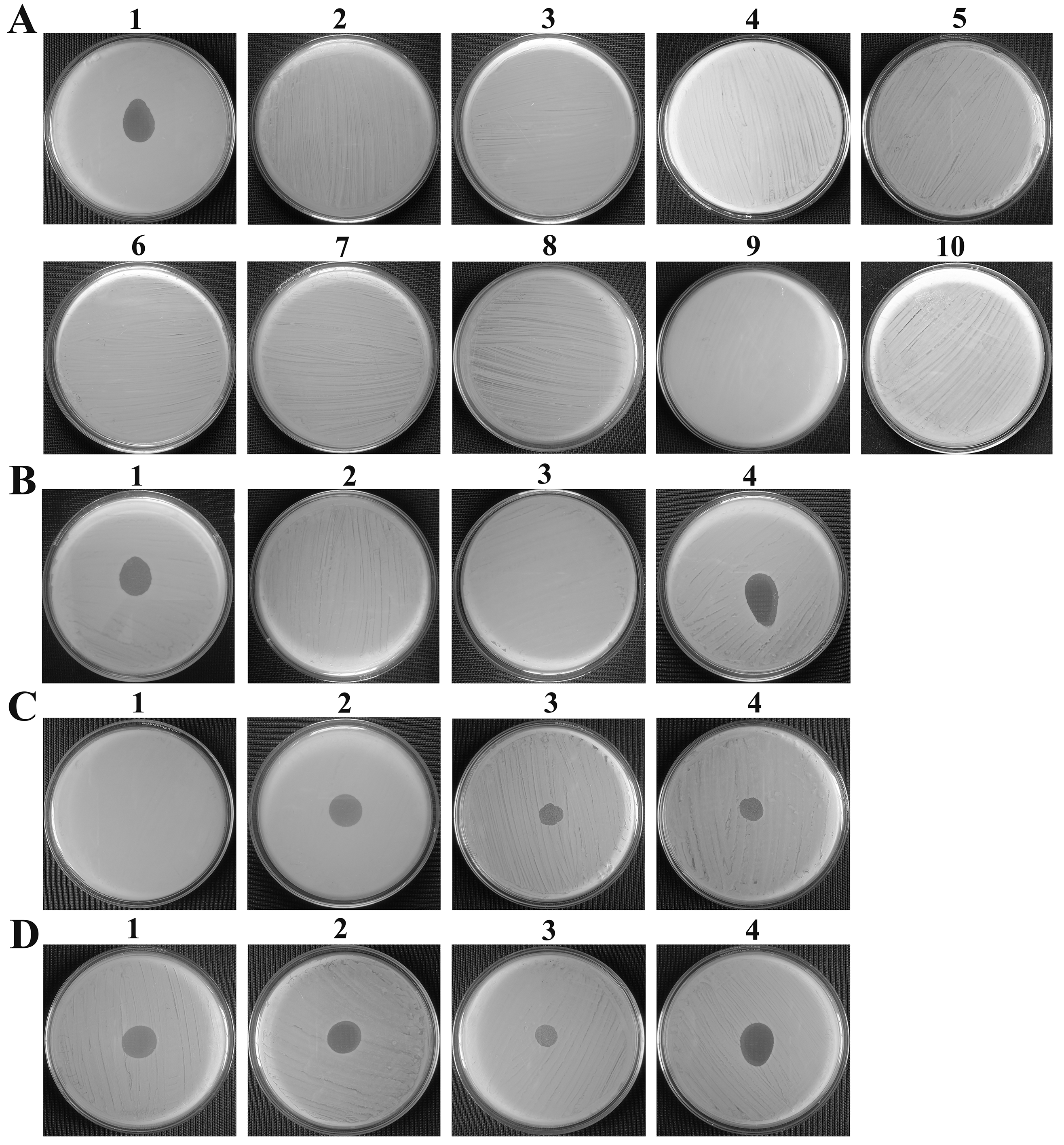

Supplement: Supplementary file 3 [file Image_1.tif]

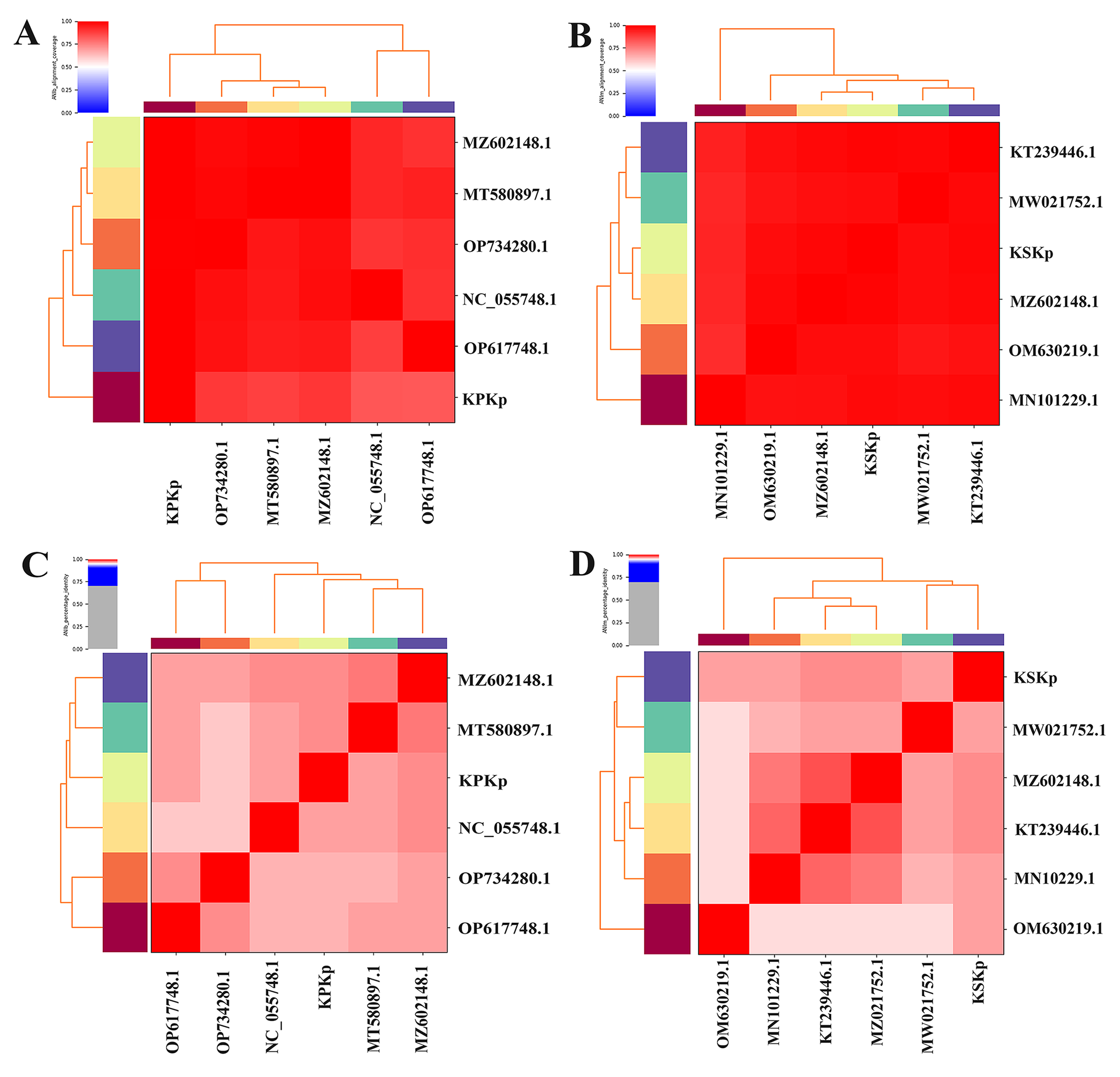

Supplement: Supplementary file 4 [file Image_2.tif]

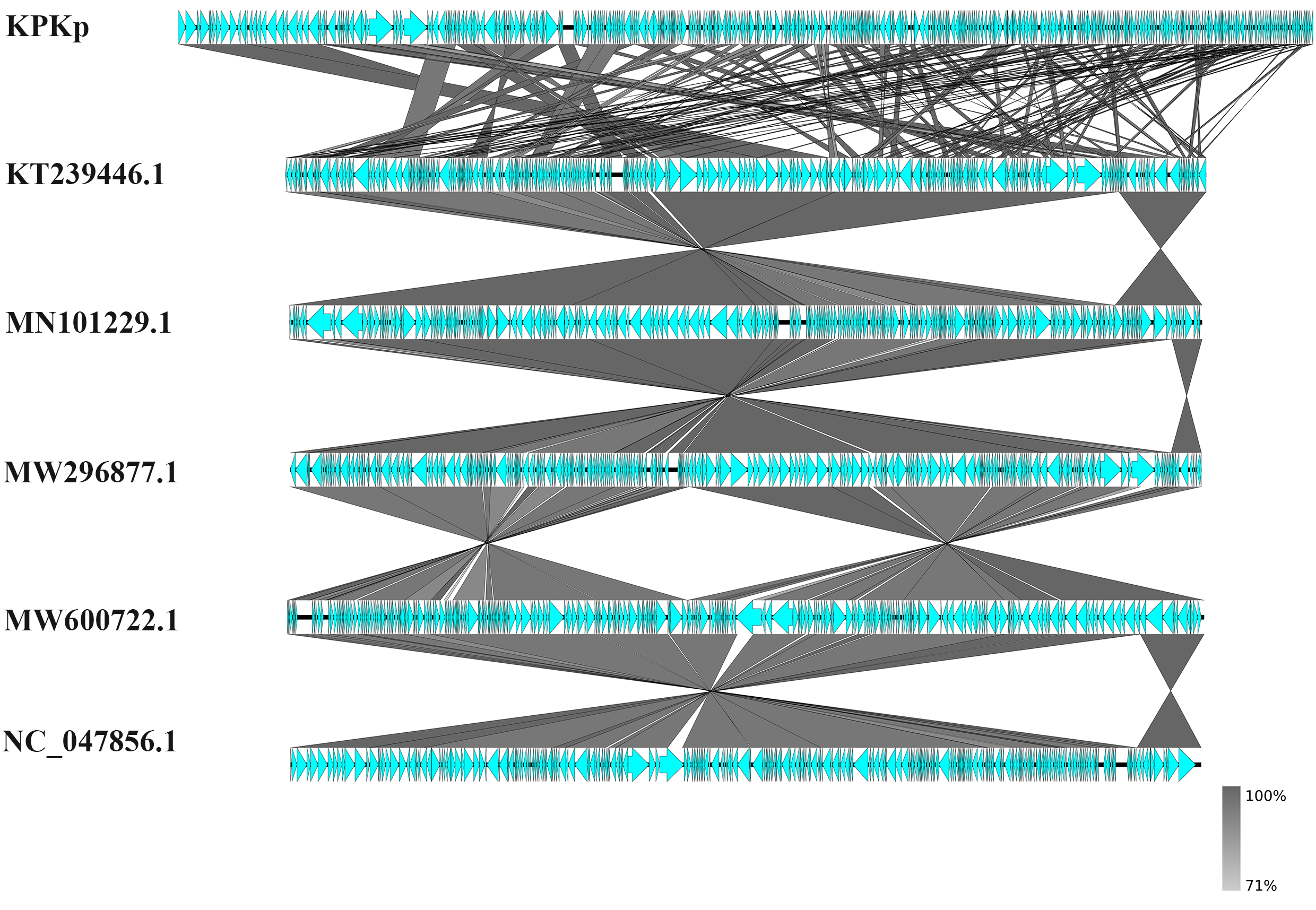

Supplement: Supplementary file 5 [file Image_3.tif]

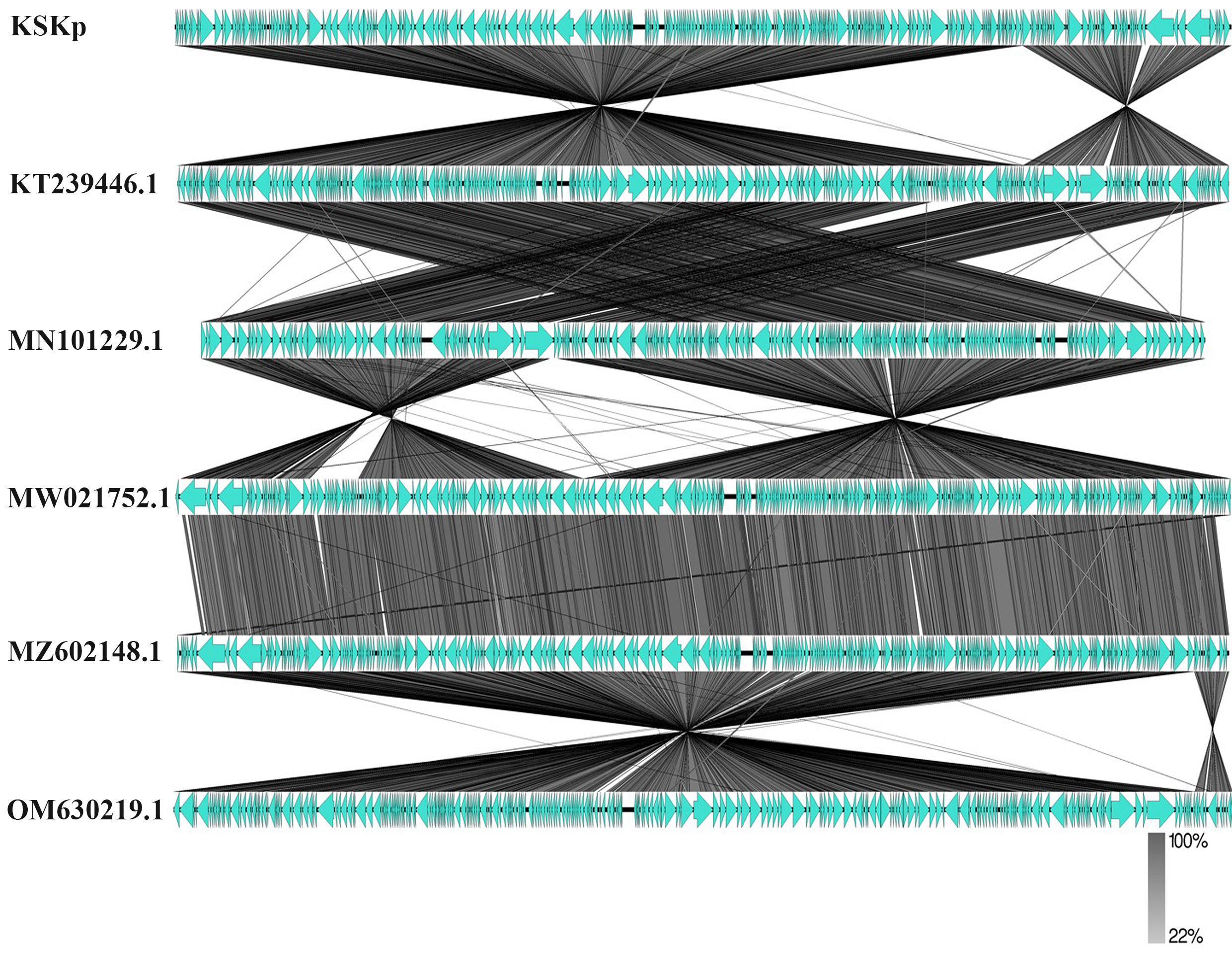

Supplement: Supplementary file 6 [file Image_4.tif]

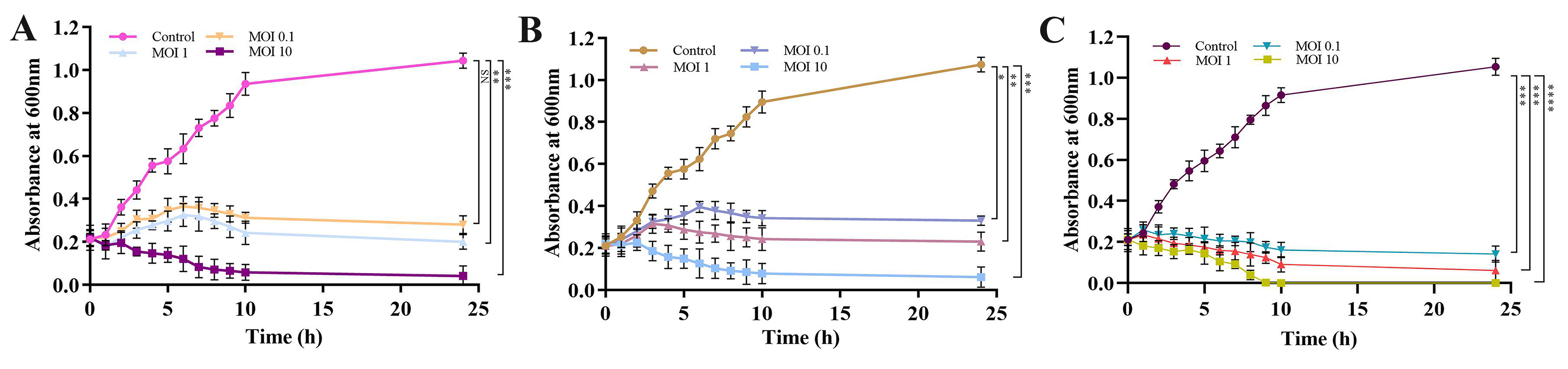

Supplement: Supplementary file 7 [file Image_5.tif]

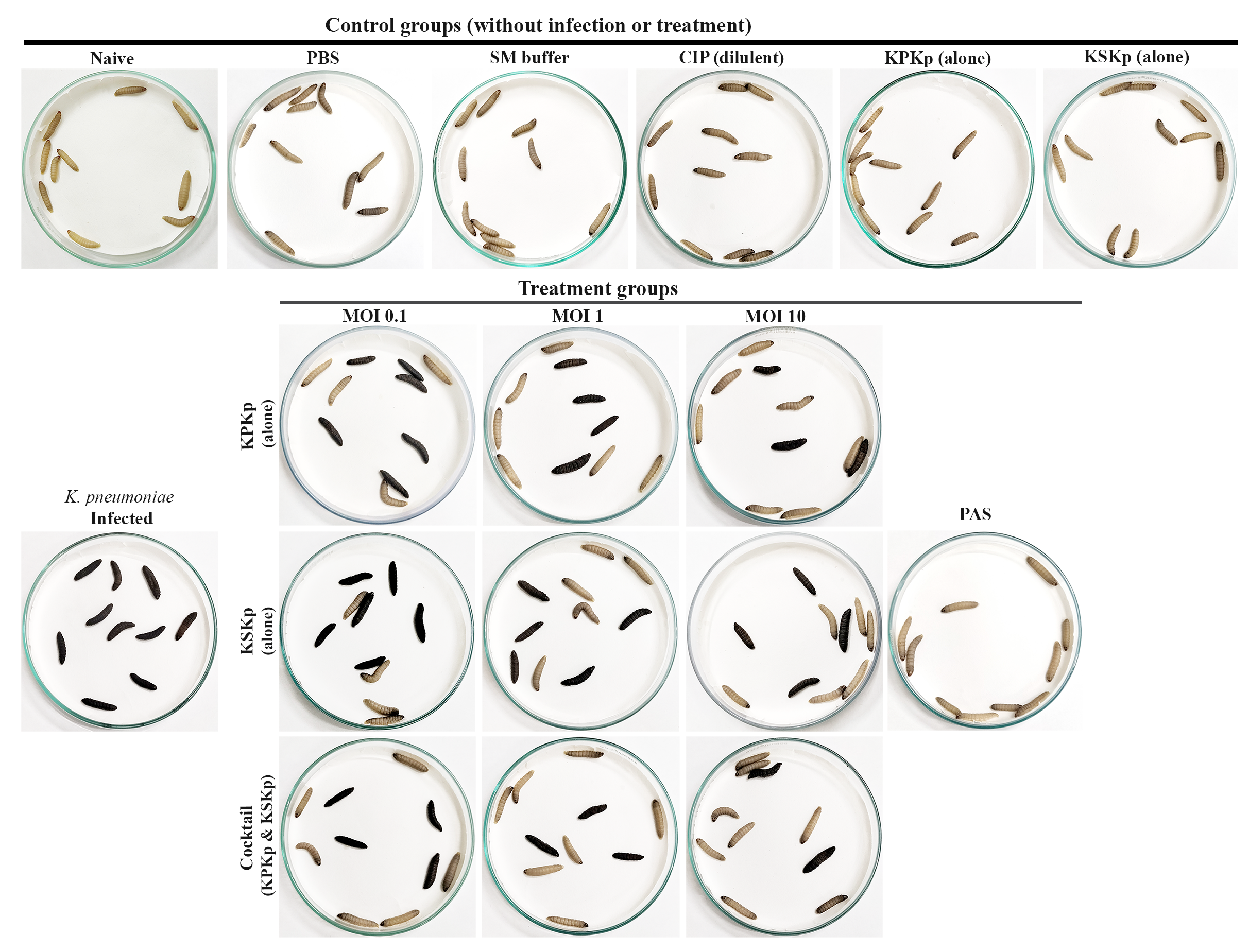

Supplement: Supplementary file 8 [file Image_6.tif]
